# Investigation of Neural Substrates of Erroneous Behavior in a Delayed-Response Task

**DOI:** 10.1523/ENEURO.0490-21.2022

**Published:** 2022-04-08

**Authors:** Soyoung Chae, Jeong-woo Sohn, Sung-Phil Kim

**Affiliations:** 1 Department of Biomedical Engineering, Ulsan National Institute of Science and Technology, Ulju-gun, Ulsan 44929, South Korea; 2 Department of Medical Science, Catholic Kwandong University, International St. Mary's Hospital, Gangneung-si, Gangwon-do 25601, South Korea

**Keywords:** error behavior, motor planning, premotor cortex, preparatory activity, selectivity, short-term memory

## Abstract

Motor cortical neurons exhibit persistent selective activities (selectivity) during motor planning. Experimental perturbation of selectivity results in the failure of short-term memory retention and consequent behavioral biases, demonstrating selectivity as a neural characteristic of encoding previous sensory input or future action. However, even without experimental manipulation, animals occasionally fail to maintain short-term memory leading to erroneous choice. Here, we investigated neural substrates that lead to the incorrect formation of selectivity during short-term memory. We analyzed neuronal activities in anterior lateral motor cortex (ALM) of mice, a region known to be engaged in motor planning while mice performed the tactile delayed-response task. We found that highly selective neurons lost their selectivity while originally nonselective neurons showed selectivity during the error trials where mice licked toward incorrect direction. We assumed that those alternations would reflect changes in intrinsic properties of population activity. Thus, we estimated an intrinsic manifold shared by neuronal population (shared space), using factor analysis (FA) and measured the association of individual neurons with the shared space by communality, the variance of neuronal activity accounted for by the shared space. We found a positive correlation between selectivity and communality over ALM neurons, which disappeared in erroneous behavior. Notably, neurons showing selectivity alternations between correct and incorrect licking also underwent proportional changes in communality. Our results demonstrated that the extent to which an ALM neuron is associated with the intrinsic manifolds of population activity may elucidate its selectivity and that disruption of this association may alter selectivity, likely leading to erroneous behavior.

## Significance Statement

Appropriate retaining of short-term memory can maximize a future reward. During retention, neurons in frontal cortex show persistent activity encoding a selection of future action, the collapse of which leads to erroneous behavior. This study addressed the underlying neural mechanism for changes of selectivity in erroneous behavior by investigating selectivity in rodent anterior lateral motor cortex (ALM) during the delayed-response task. We found that the stronger a neuron’s activity was coupled to an intrinsic shared space of ALM, the greater its selectivity was. Also, changes in selectivity during erroneous behavior were related to changes in coupling strength. Our work suggests that proper association with the shared space is key to orchestrating ALM neuronal activities for accurate planning for upcoming movement.

## Introduction

Appropriate motor planning is essential to accurate motor control. Neurons in motor cortex modulate their activity for motor planning before movement onset (Tanji and Evarts, 1976; Weinrich et al., 1984). This preparatory activity contains information on forthcoming movement such as reaction time (Riehle and Requin, 1989; Churchland and Shenoy, 2007). Similar to motor cortical preparatory activity shown in nonhuman primates, anterior lateral motor cortex (ALM), which is a central part of motor planning circuits in mouse, shows selective firing activities (i.e., termed as selectivity) depending on the direction of upcoming movements (Li et al., 2015). Neural circuits involving ALM neurons that generate selectivity during movement preparation have been investigated using a delayed-response task where a sensory cue informs animals which direction to lick after delay (Chen et al., 2017; Guo et al., 2017; Gao et al., 2018; Wang et al., 2021). For example, disruption of selectivity in ALM by photoinhibiting relevant neural circuits leads to failure of short-term memory retention. Thus, proper maintenance of selectivity is necessary for ALM to link past sensory cue and future action.

Even after learning a delayed-response task, however, animals often perform the task incorrectly without external perturbation. Such erroneous behavior is likely to be associated with error in motor planning, potentially attributed to several hypothetical sources. For instance, a received sensory cue could be misrepresented in neurons participating in movement preparation (Panzeri et al., 2017). Or the stochastic nature of the evolution of neural states underlying motor cortical activity can drive neural states toward a wrong subspace by chance (Inagaki et al., 2019). While these accounts are plausible and worth exploring, a simpler starting point to investigate neural substrates of erroneous behavior would be examining possible sources that underpin changes in the selectivity of neurons, as selectivity has been shown to be substantially disrupted when movement error ensues (Li et al., 2015) .

Therefore, the present study aims to investigate neural substrates of erroneous behavior in a delayed-response task by focusing on neural determinants of the disruption of selectivity during motor planning. To this end, we analyze ALM activity in three folds. First, at a single neuronal level, we examine how the selectivity of single ALM neurons is disrupted for erroneous behavior. Second, at a neuronal population level, we investigate whether there is a collective pattern in the disruption of selectivity by inspecting an intrinsic manifold shared by population (i.e., the shared space; Athalye et al., 2017). We employ factor analysis (FA) to infer the shared space from observed ALM population activity. Third, by integrating both single neuron and population levels, we associate individual neuronal activities with the shared space and analyze how these associations are altered during erroneous behavior. As FA allows the decomposition of individual neuronal activities into shared and private signals (Athalye et al., 2017), where the shared signal reflects the portion of a neuronal activity generated from latent factors in the shared space, the analysis of the shared signal would reveal how disruption of population-level activity connects to that of individual neuronal activity. Specifically, we investigate how the selectivitiy of a single neuron is related to the shared space and whether such a relationship is altered for erroneous behavior.

At the single neuronal level, we observed alternations in the selectivity during erroneous motor planning. We observed a false drive of selective firings of ALM neurons, resulting in increases in the selectivity of those neurons that were less selective in preparation of correct behavior and vice versa. At the population level, we confirmed that movement direction information was inadequately represented in the shared space during erroneous motor planning. Finally, by associating the selectivity of single neurons with the shared space, we found that the selectivity of single neurons was positively correlated with the variance of neuronal activity accounted for by the shared space (i.e., termed as communality), which showed that neurons more strongly tied to the shared space tended to exhibit greater selectivity. Such correlations disappeared when the mice licked to the incorrect direction. We found that changes of selectivity from correct to incorrect trials were positively correlated with changes of communality from correct to incorrect trials during the delay period. It suggests that erroneous behavior may be caused by both the decreased selectivity of originally more selective neurons and the increased selectivity of originally less selective neurons, which seems to occur in relation to changes in those neurons’ coupling to the shared space, especially during motor planning.

## Materials and Methods

### Datasets

**Figure 1.**
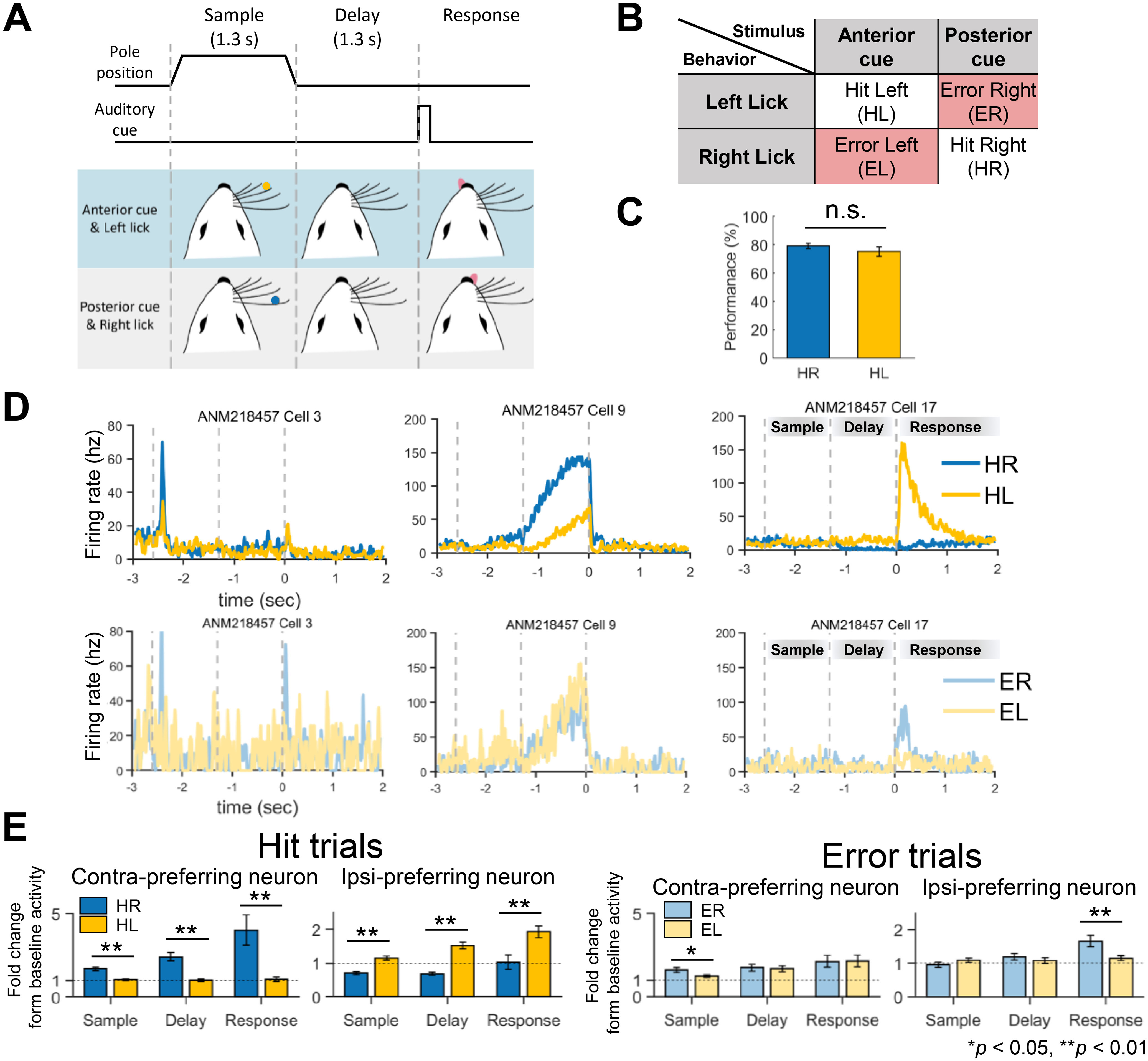
Disruption of selective ALM activity tuned to the licking direction during erroneous behavior. ***A***, A schematic diagram for the tactile delayed-response task. In the sample period, a tactile cue represented as a pole position at either anterior or posterior whiskers was given for 1.3 s to indicate the upcoming reward (water) direction. The anterior cue was associated with the left direction whereas the posterior cue was with the right. Mice should not move but wait during a 1.3-s delay period and began to lick toward either the left or right direction after hearing an auditory go cue. ***B***, Four possible behavioral results from the delayed-response task depending on the match between the tactile cue (anterior vs posterior) and licking direction (left vs right): HL, ER, EL, and HR. ER (left) denotes erroneous movement to the left (right) given a right-directing (left-directing) cue. ***C***, No significant difference in the behavioral performance of the tactile delayed-response task between licking directions (*p *>* *0.1, two-tailed paired *t* test, *n* = 22). Error bars, SEM across the mice. n.s.: not significant. ***D***, Examples of the selective firing activities (i.e., selectivity) of three representative ALM neurons when mice performed the tactile delayed-response task, for each of the four cases of behavioral outcomes (HL, HR, ER, and EL). Each neuron showed peak activity in a particular period when the task goal (left or right direction) agreed with its selectivity (left or right) and when mice behaved correctly (HL and HR). This selective firing activity became more ambiguous when mice behaved wrongfully (ER and EL). Also, differences in activities between HR and HL in a particular period were large in the hit trials (top), which was less apparent in the error trials (bottom). ***E***, Differences in the firing activity of the ALM neurons showing selectivity between licking directions. Contra-preferring neurons denote the ALM neurons with selectivity to the right direction (note: left ALM neurons were recorded) and ipsi-preferring neurons do for the left direction. Differences in firing activities of these neurons between licking directions were shown in the hit and error trials, based on the fold changes from baseline activity (*p <* *0.05, **p <* *0.01, Bonferroni-corrected *post hoc* test). While the neurons exhibited significant differences between the directions for every period in the hit trials (left), such differences mostly disappeared in the error trials (right). Error bars, SEM across the neurons.

In this study, we analyzed two open datasets (Li et al., 2014; Chen et al., 2016) that contained the same experimental data in a total of 38 mice (26 males and 12 females, ages >P60; P: Postnatal day). Action potentials (spikes) were simultaneously recorded in left ALM with silicon probes (part #A4x8-5 mm-100-200-177, NeuroNexus). The datasets are publicly available online at the Collaborative Research in Computational Neuroscience website (http://crcns.org), contributed by the Svoboda laboratory. A detailed description of the procedure to collect data can be found in previously published work (Li et al., 2015, 2016). In brief, the mice were trained to sense the contact position of a pole (anterior or posterior) in their whiskers to perform a tactile delayed-response task (Fig. 1*A*). At the beginning of each trial of the task, a pole touched the whisker of the mice for 1.3 s (sample period), cueing the direction of an upcoming reward (left or right). After the pole was detached from the whisker, the mice waited for another 1.3 s (delay period), then executed a licking movement (response period; Fig. 1*A*). The mice received a water reward if they licked to the right provided that the pole had touched the posterior part [called a hit right (HR) trial], or to the left provided that it had touched the anterior part of the whisker [called a hit left (HL) trial]. A trial ended with no reward if the mice licked either to the left given the posterior cue [called an error right (ER) trial] or to the right given the anterior cue [called an error left (EL) trial; Fig. 1*B*]. On average, each mouse performed 4.84 sessions for multiple days, where each session consisted of 100.43 trials of HR, HL, ER, and EL. Extracellular traces were recorded from left ALM and bandpass filtered (300–6000 Hz). A spike was extracted from the filtered trace by visual inspection with a spike width calculated as a trough-to-peak interval in the average spike waveform (Guo et al., 2014). Units with spike width <0.35 ms were defined as fast-spiking GABAergic (FS) neurons (196/2420) and units with spike width >0.45 ms as putative pyramidal neurons (2135/2420). Units with intermediate values (0.35–0.45 ms) were excluded from our analyses (89/2420).

### Neuronal firing rates

The firing rate of a neuron was calculated by counting spikes within a nonoverlapping 100-ms bin. We also defined a firing rate change for each period (sample, delay, and response) as the mean firing rate in each period divided by the mean firing rate in baseline (0.3–0 s before tactile cue onset; Fig. 1*E*). Note that no firing rate change was calculated for those neurons which did not fire during baseline.

### Selectivity

ALM neurons reveal selectivity that characterizes differential firing rates depending on licking directions (Li et al., 2015, 2016; Guo et al., 2017; Economo et al., 2018; Gao et al., 2018; Inagaki et al., 2018, 2019). We classified a neuron as ipsi-preferring if its firing rate was significantly higher in the HL than in the HR trials, contra-preferring if vice versa, or nonselective if no significant difference was found (*p *<* *0.01, one-tailed Mann–Whitney test). We conducted this classification of neurons independently within each period.

We defined the selectivity of an ipsi-preferring, or contra-preferring, neuron in a given similar to the previous study (Inagaki et al., 2018):

(1) $$\text{Selectivity}=2\text{ × }\frac{fr_{HR}-fr_{HL}}{Max\left(fr_{HR}\right)+Max(fr_{HL})},$$

where *fr_HR_* (*fr_HL_*) and *Max*(*fr_HR_*) (*Max*(*fr_HL_*)) denote the mean and maximum firing rates across the HR trials (HL trials), respectively. From Equation 1, contra-preferring neurons should have positive selectivity whereas ipsi-preferring neurons should have negative one. Normalization in Equation 1 was necessary for generalizing and comparing the selectivity across neurons regardless of sessions. In normalization, we divided the difference in firing rates between HR and HL trials by maximum firing rate during HR and HL trials. Most of the selectivity had value between −0.5 and 0.5, we multiplied two to set the selectivity value between −1 and 1. Note that the maximum values are only for the period under consideration (sample, delay, and respond period each), and we used mean firing rate in the period (averaged across time points) and calculated the selectivity.

Then, we estimated the selectivity of a neuron from data in the same way as the previous studies (Li et al., 2015; Inagaki et al., 2018). Specifically, we randomly sampled firing rates from 30% of the HR and HL trials and calculated $fr_{HR}-fr_{HL}$ in each period. We repeated this calculation in Equation 1 1000 times and obtained the mean value, which was applied to Equation 1 to compute selectivity. We also estimated the selectivity of a neuron in the error trials with the same procedure, but by replacing the HR and HL with ER and EL trials, respectively (i.e., neurons selective in ER: $fr_{ER}>fr_{HL}$). Note that we used Mann–Whitney test instead of *t* test to classify an ipsi-preferring or contra-preferring neuron in the error trials.

### Factor Analysis (FA)

We used FA to infer a shared space, an intrinsic manifold shared by neuronal population activity (Churchland et al., 2010; Everett, 2013; Athalye et al., 2017, 2018; Wei et al., 2019). Unlike other dimensionality reduction techniques such as principal component analysis (PCA), FA focuses on finding latent variables (i.e., factors) that best describe covariance between neurons (Byron et al., 2009; Athalye et al., 2017, 2018). Moreover, FA decomposes the firing activity of a neuron into a shared signal, which is accounted for by population-shared latent variables, and a private signal, which is independent of latent variables.

Before applying FA to population activity, we first trimmed firing activity data. So, to detect and remove those neurons which did not exhibit action potentials because of unstable recordings, we excluded neurons that were silent for >50% of the trials. After this process, the data of 63 sessions in 22 mice out of 184 sessions in 38 mice were used for FA. We applied FA to the firing rate data of a neuronal population in each period. As the previous study on the same data showed that ALM neuronal firing activities during the delay period could be well represented on a two-dimensional space (Inagaki et al., 2018), we also determined the number of factors as two in our analyses. The previous study on the same task paradigm showed that two modes capture over 60% across-trial variance in ALM activities. (Inagaki et al., 2018). We calculated the mean variance explained by principal components after 100 random subsampling of hit trials to match the number of hit trials and the number of error trials. We observed two principal components explained similar level of variance of our data both in the hit and error trials (hit trials, sample: 62.22 ± 0.0069%, delay: 65.35 ± 0.0056%, response: 64.34 ± 0.0067% mean ± SEM across subsampling iterations; error trials, sample: 61.55 ± 1.40%, delay: 64.19 ± 1.50%, response: 62.84 ± 1.35%; mean ± SEM across sessions). Note that mean variance explained in the hit trials is mean of variance explained across iterations of subsampling the hit trials. Because mean variance explained is over 60% regardless of behavioral results (hit and error) and periods (sample, delay, and response), we decided to fix K as two.

A specific procedure to conduct FA on ALM data are as follows. Let $x\in R^{N}$ be a vector of the firing rates of *N* neurons and $z\in R^{k}$ be a *K*-dimensional random vector (*K *<* N*) following a multivariate normal distribution such as:

(2) z ∼ N(0,I).

FA assumes that x is generated from z by a linear model:

(3) $$x∼N\left(\mu +Uz,UU^{T}+\psi \right),$$

where $\mu \in R^{N}$ is a vector of the mean firing rates of *N* neurons, $U\in R^{N\times k}$ is a factor loading matrix illustrating a generative relationship from *z* to *x* and $\psi \in R^{N\times N}$ is a covariance matrix of residuals. We form a vector of the shared signals of *N* neurons, $x^{shared}=Uz$, and that of the private signals of *N* neurons $x^{private}=x-\mu -Uz$. Each vector follows a multivariate normal distribution:

(4) $$x^{shared}∼N(0,\;{\text{Σ}}^{shared})$$

(5) $$x^{private}∼N(0,\;{\text{Σ}}^{private}),$$

where ${\text{Σ}}^{shared}=UU^{T}$ and ${\text{Σ}}^{private}=\text{Ψ}$. We can decompose *x* and its covariance as:

(6) $$x=\mu +x^{private}+x^{shared}$$

(7) $${\text{Σ}}^{total}={\text{Σ}}^{private}+{\text{Σ}}^{shared},$$

where ${\text{Σ}}^{total}$ denotes the covariance matrix of *x*. The factor loading matrix *U* is estimated by the expectation-maximization (EM) algorithm (Dempster et al., 1977; Athalye et al., 2017, 2018).

### Representation of licking directions in the shared space

We evaluated the representation of licking directional information in the shared space. Let *Z* be the matrix of the factor scores from every hit trial, *Z* ∈ R*^T^*^x^*^K^*, where *T* is the number of the hit trials, including both the HR and HL trials and *K* is the number of factors. We assigned a factor score vector of each trial (i.e., each row of *Z*) to one of the two clusters corresponding to the licking direction. Then, we measured how well the two clusters were separated using the Fisher ratio (FR) given by:

(8) $$\text{FR}\left({\text{LV1}}_{\text{HR}},{\text{ LV1}}_{\text{HL}}\right)=\frac{{\left(E{\left[LV1\right]}_{HR}-E{\left[LV1\right]}_{HL}\right)}^{2}}{Var{\left[LV1\right]}_{HR}+Var{\left[LV1\right]}_{HL}},$$

where LV1 is the first latent variable (i.e., the first factor) and *E*[·]*_HR_*_/_*_HL_* and *Var*[·]*_HR_*_/_*_HL_* represent its expected value and variance over the HR/HL trials, respectively. We also calculated the FR(LV2_HR_, HV2_HL_) for the second latent variable (LV2, the second factor) in the same way. The higher the FR is, the more the two clusters are separated. We repeated the same separability analysis for the error trials, where we assigned each factor score vector to one of the two clusters corresponding to the tactile cue instead of actual licking direction.

To establish a statistical criterion for determining whether the latent variables contained licking directional information, we calculated a random FR by randomizing directional information. We shuffled directional information of all the hit trials, clustered latent variables accordingly, and measured the FR between the clusters. We repeated this procedure multiple times to establish a distribution of the random FR.

We validated the reliability of licking directional representations in the shared space via a train-and-test scheme. In this scheme, we first built a shared space using the first half of the hit trials such that the first half was used as training data. Then, we projected the firing rate data of the second half of the hit trials or those of the error trials onto that shared space such that these remaining data were used as testing data. The projection of a testing firing rate vector of the second half of the hit trials or the error trials, *x*, onto the shared space was conducted by estimating a corresponding shared signal ( ${\hat{x}}^{shared}$) and a factor score vector ( $\hat{z}$) as following (Athalye et al., 2017):

(9) $${\hat{x}}^{shared}=E\left[x^{shared}\text{|}x]=E[Uz|x\right]=UU^{T}{\left(UU^{T}+\text{ψ}\right)}^{-1}(x-\mu )$$

(10) $$\hat{z}={\left(U^{T}U\right)}^{-1}U^{T}{\hat{x}}^{shared},$$

where μ, U and ψ were estimated from the training data. Here, we denote a set of estimated factor score vectors from the second half of the hit trials as HIT*_test_* and that from the error trials as ERR*_test_*. We also repeated the same projection using the training data and denote a set of factor score vectors from the first half of hit trials as HIT*_train_*.

Then, we measured a similarity between HIT*_train_* and HIT*_test_* or between HIT*_train_* and ERR*_test_* to assess the reliability of directional representations in the shared space. To this end, we divided the factor score vectors in each of HIT*_train_*, HIT*_test,_* and ERR*_test_* into two clusters, respectively, according to the cue information (i.e., cued direction). Then, we calculated the FR between the two clusters of HIT*_train_* and HIT*_test_* or HIT*_train_* and ERR*_test_*, that were assigned to the same cue. Similarly, we calculated the FR between the clusters assigned to the opposite cue. If the shared space remained consistent between training and testing, HIT*_train_* and HIT*_test_* would form similar clusters and the two clusters assigned to the same cue would be overlapped, resulting in a smaller FR. On the other hand, the two clusters assigned to the opposite cue would be apart from each other with a larger FR. We also examined whether this examination was held for ERR*_test_*. For reference, we calculated the FR between the two clusters of HIT*_train_* and compared other FR values to it. Pairwise comparisons between the reference and the other four FR values were performed: (1) FR(HIT*_train_*, HIT*_test_*) for same cue, (2) FR(HIT*_train_*, HIT*_test_*) for opposite, (3) FR(HIT*_train_*, ERR*_test_*) for same, and (4) FR(HIT*_train_*, ERR*_test_*) for opposite.

### Selectivity of shared signals

To investigate whether the shared signal of each ALM neuron exhibited selectivity, we estimated selectivity of shared signals in the same way as firing rates (see above), by replacing firing rates in Equation 1 with shared signals, $x^{shared}$. Hereafter, we denote the selectivity of the shared signal of a neuron as Sel_SH_.

### Reversed firing modulation with erroneous behavior

We assessed whether the selectivity of ALM neurons vanished or was reversed for erroneous behavior by comparing individual neuronal firing activities between the error trials and the hit trials. Among ipsi-preferring, or contra-preferring, neurons, we examined whether there existed neurons that reversed their firing modulation with erroneous behavior by showing significantly higher firing rates in a nonpreferred cue trial than in a preferred cue trial during the erroneous behavior (Mann–Whitney test, *p *<* *0.05).

Then, we examined whether such neurons reversed their firing rates together with other neurons or independently during the task. To this end, we calculated trial-by-trial correlations between the firing rates of all possible pairs of those neurons which showed reversed firing modulation, in each of the error trials and the hit trials. Then, we evaluated whether correlations were different or not between the hit and error trials using Wilcoxon signed-rank test. If the correlations in the error trials remained unchanged compared with the hit trials, it would indicate that the neurons reversed firing modulation collectively during the error trials.

### Analysis of generative relations from latent variables to shared signals

We further analyzed how a generative relation from latent variables to the shared signals of individual neurons was altered between the hit and error trials. Since this generative relation, described by the factor loading matrix (*U*), could be altered by changes in *U*, changes in latent variables (*z*), or changes in both *U* and *z*, we examined the effect of *U* or *z* on the selectivity of shared signals (Sel_SH_). To this end, we reconstructed shared signals in two ways. First, we reconstructed shared signals through $x^{shared}=Uz$ with *U* estimated using the data in the hit trials and *z* inferred using the data in the error trials. Second, we repeated the same reconstruction with *U* estimated using the error trials and *z* inferred using the hit trials. In each session, we calculated selectivity of shared signals reconstructed in either the first or the second way above (reconstructed Sel_SH_). We also calculated selectivity of shared signals originally generated using the data in the hit trials (original Sel_SH_). Collecting these selectivity values from all sessions, we calculated correlations between original Sel_SH_ and reconstructed Sel_SH_ for each way of reconstruction. If reconstructed Sel_SH_ in the first way was positively correlated with original Sel_SH_, it would indicate that *z* remained similar across the hit and error trials and possible changes in selectivity of neurons might be attributed to changes in *U*. On the other hand, if reconstructed Sel_SH_ in the second way was positively correlated with original Sel_SH_, it would indicate that *U* remained relatively similar across the hit and error trials and possible changes in selectivity of neurons might be attributed to changes in *z*.

### Communality

We employed communality as a metric to measure how much the firing activity of a single neuron was explained by the shared space. Specifically, the communality of a neuron was calculated as the sum of squared factor loadings associated with the neuron, thus representing how much variance of the neuron’s firing rate was accounted for by the latent variables. The *i*^th^ neuron’s communality was calculated by:

(11) $$Communality_{neuron_{i}}=u_{i1}^{2}+u_{i2}^{2},$$

where *u_i_*_1_ and *u_i_*_2_ constitute the *i*^th^ row of the factor loading matrix *U* in Equation 3.

After calculating the communality of every neuron, we assessed a relationship between the selectivity and communality of individual neurons using the linear regression analysis, where a dependent variable and an independent variable were the selectivity and communality of each neuron, respectively. Statistical significance of linear regression was evaluated by the *F* test.

## Results

### ALM neuronal selectivity changed when mice licked to wrong direction

We first verified that a success rate of the tactile delayed-response task was not different between licking directions across the sessions selected for FA (63 sessions): the mean and standard error of the success rate was 79.17 ± 0.08% for the HR trials and 75.18 ± 0.15% for the HL trials, respectively (*p *=* *0.28, two-tailed paired *t* test; Fig. 1*C*).

During the task, many ALM neurons showed selectivity in specific periods (see examples in Fig. 1*D*). While the firing rates of selective neurons were obviously higher for their preferred cue than nonpreferred ones in the hit trials (Fig. 1*E*, left), such differences were largely absent in the error trials (Fig. 1*E*, right). Notably, the firing rates of ipsi-preferring neurons were even higher in the ER than in the EL trials during the response period (Fig. 1*E*, right).

**Figure 2.**
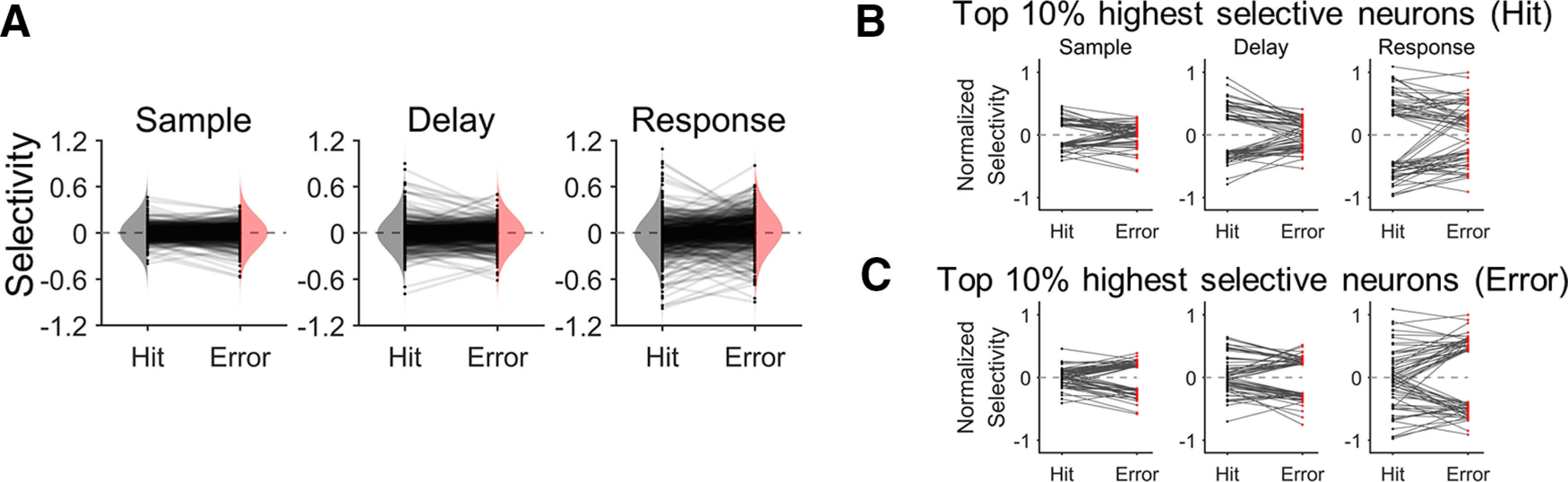
Changes in selectivity of ALM neurons between correct and erroneous behavior. ***A***, Distributions of selectivity in the correct (hit) and erroneous (error) trials for each period (sample, delay, and response). Black dots represent individual neuronal selectivity in the hit and the error trials. Gray lines connecting each pair of the black dots indicate the selectivity change of the corresponding neuron between the hit and error trials. The vertically oriented shadings indicate the sample distributions of selectivity for hit (gray) or error (pink) trials, respectively. While individual neuronal selectivity was decreased or increased across the hit and error trials, there was no significant difference in the distribution of the selectivity between the hit and error trials (K-S test, *p *>* *0.05) for every period. ***B***, 10% of the ALM neurons, marking the top 5% contra-preferring and the top 5% ipsi-preferring selectivity in the hit trials (black dots), significantly decreased their selectivity in the error trials (red dots) for every period (one-tailed paired *t* test, *p *<* *10^−6^). The gray lines indicate selectivity changes between hit and error trials of each neuron. ***C***, 10% of the ALM neurons, marking the top 5% contra-preferring and the top 5% ipsi-preferring selectivity in the error trials (black dots), significantly decreased their selectivity in the hit trials (red dots) for every period (one-tailed paired *t* test, *p *<* *0.01). The gray lines indicate selectivity changes between hit and error trials of each neuron.

Next, we inspected changes of selectivity between the hit and error trials. The Kolmogorov–Smirnov test (K-S test) revealed no significant difference in the overall distributions of selectivity between the hit and error trials (*p*s > 0.05 for every period; Fig. 2*A*). Thus, it confirmed that selectivity did not disappear during the error trials. Rather, we observed that neurons with higher selectivity in the hit trials tended to show reduced selectivity in the error trials whereas those with lower selectivity in the hit trials tended to show increased selectivity in the error trials (Fig. 2*A*). To examine these observations, we selected neurons showing selectivity within the top (showing high selectivity in HR trials) and bottom (showing high selectivity in HL trials) 5% of the selectivity distribution in the hit trials and tracked their selectivity in the error trials. The absolute values of selectivity of these neurons significantly decreased from the hit to error trials (*p*s < 10^−6^ for every period, one-tailed paired *t* test; Fig. 2*B*). Similarly, we conducted the same analysis in the opposite direction – selecting neurons with the top (showing selectivity in ER trials) and bottom (showing selectivity in EL trials) 5% selectivity in the error trials and tracking their selectivity in the hit trials, and observed significant decreases of the absolute selectivity from the error to hit trials (*p*s < 0.01 for every period, one-tailed paired *t* test; Fig. 2*C*). We found that neurons selective during the hit trials decreased their selectivity in the error trials (*p*s < 0.01 for every period, one-tailed paired *t* test) and neurons selective during the error trials also decreased selectivity in the hit trials (*ps *<* *0.05 for every period, one-tailed paired *t* test). Thus, relatively less selective neurons during the hit trials could gain more selectivity during the error trials, indicating that those neurons that were significantly selective during the hit trials decreased selectivity during the error trials, and those neurons that were significantly selective during the error trials also decreased selectivity during the hit trials. We explored this pattern in terms of neuronal relations to the shared space in the following analyses.

### Reversed firing modulation with erroneous behavior

**Figure 3.**
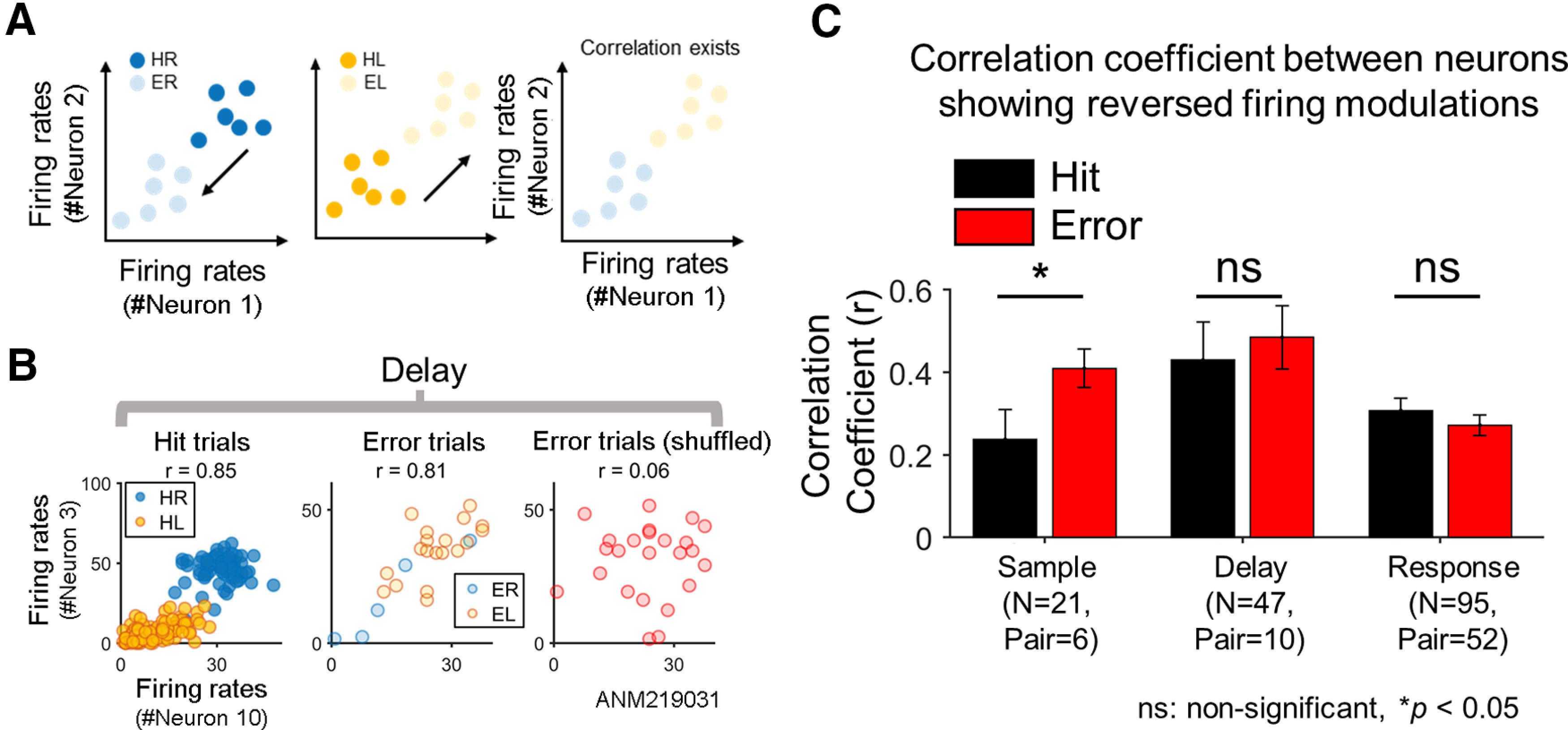
Reversed firing modulation of ALM neurons. ***A***, The schematic diagram illustrating reversed firing modulation (see Materials and Methods). If (hypothetic) neurons decrease firing rates in the error trials in response to a preferred cue that originally increases the firing rates in the hit trials and vice versa, neurons are deemed to exhibit reversed firing modulation. For example, with reversed firing modulation, neurons that show higher firing rates for correct right licking (HR) than for correct left licking (HL) would decrease firing rates in response to a right directional cue for erroneous left licking (ER; left) while increase firing rates in response to a left directional cue (EL; middle). If two neurons with similar selectivity exhibit reversed firing modulation, their firing rates would be correlated even over the error trials as well as over the hit trials (right). ***B***, Examples of correlated firing rates of two ALM neurons showing reversed firing modulation. In the hit trials, two contra-preferring neurons (neurons #3 and #10, session ALM219031) similarly increased firing rates when the posterior cue was given, showing a high correlation (*r *=* *0.85) between their firing rates over the hit trials (left). But in the error trials, both neurons increased firing rates when the anterior cue was given, such as ipsi-preferring neurons, showing again a high correlation (*r *=* *0.81) over the error trials (middle). Yet, such a correlation disappeared in the error trials when the trial order was shuffled (right). ***C***, Correlations between neurons showing reversed firing modulation. Pearson correlation coefficient was calculated between all pairwise combinations of the neurons showing reversed firing modulation (for the criterion to determine a neuron with reversed firing modulation, see Materials and Methods) for the hit and error trials, respectively, in each period. The average correlation coefficient was not significantly different between the hit and error trials (two-tailed paired *t* test, *p *>* *0.1) in the delay and response periods, or greater over the error trials than over the hit trials in the sample period (one-tailed paired *t* test, sample: *p *<* *0.05). *N* denotes the total number of neurons showing reversed firing modulation summed over the sessions. *Pair* denotes the sum of the number of all possible pairs of such neurons calculated session-wise (e.g., if *N *=* *2 in session 1 and *N *=* *3 in session 2, then *Pair* = *_2_*C_2_ + *_3_*C_2_ = 5). Note that *N* and *Pair* should remain the same across the hit and error trials in a given period. Error bars, SEM across pairs. n.s.: not significant.

We inspected whether there was a set of neurons collectively showing reversed firing modulation between the error and the hit trials (see the definition of reversed firing modulation in Materials and Methods), as conceptually illustrated in Figure 3*A* (e.g., contra-preferring neuron in the hit trials changes to ipsi-preferring neuron in the error trials). In effect, among selective neurons, some neurons jointly showed reversed firing rate modulation in the error trials and such joint reversal of firing modulation disappeared when directional information was shuffled across the neurons (for example, see Fig. 3*B*). We calculated pairwise correlations between neurons showing reversed firing modulation in contra-preferring and ipsi-preferring neurons respectively. Note that we pooled the correlation coefficients of contra-preferring and ipsi-preferring neurons together and conducted statistical test because of small number of sample size. As a result, we found that the correlation coefficients of neurons showing reversed remained unchanged or even greater in the error trials (*p *<* *0.05 in sample; *p*s > 0.05 in delay and response period, sign rank-test; Fig. 3*C*). These results support that the selectivity was reversed in a number of ALM neurons followed by behavioral error.

### Representation of licking directions in the shared space was disrupted for erroneous behavior

**Figure 4.**
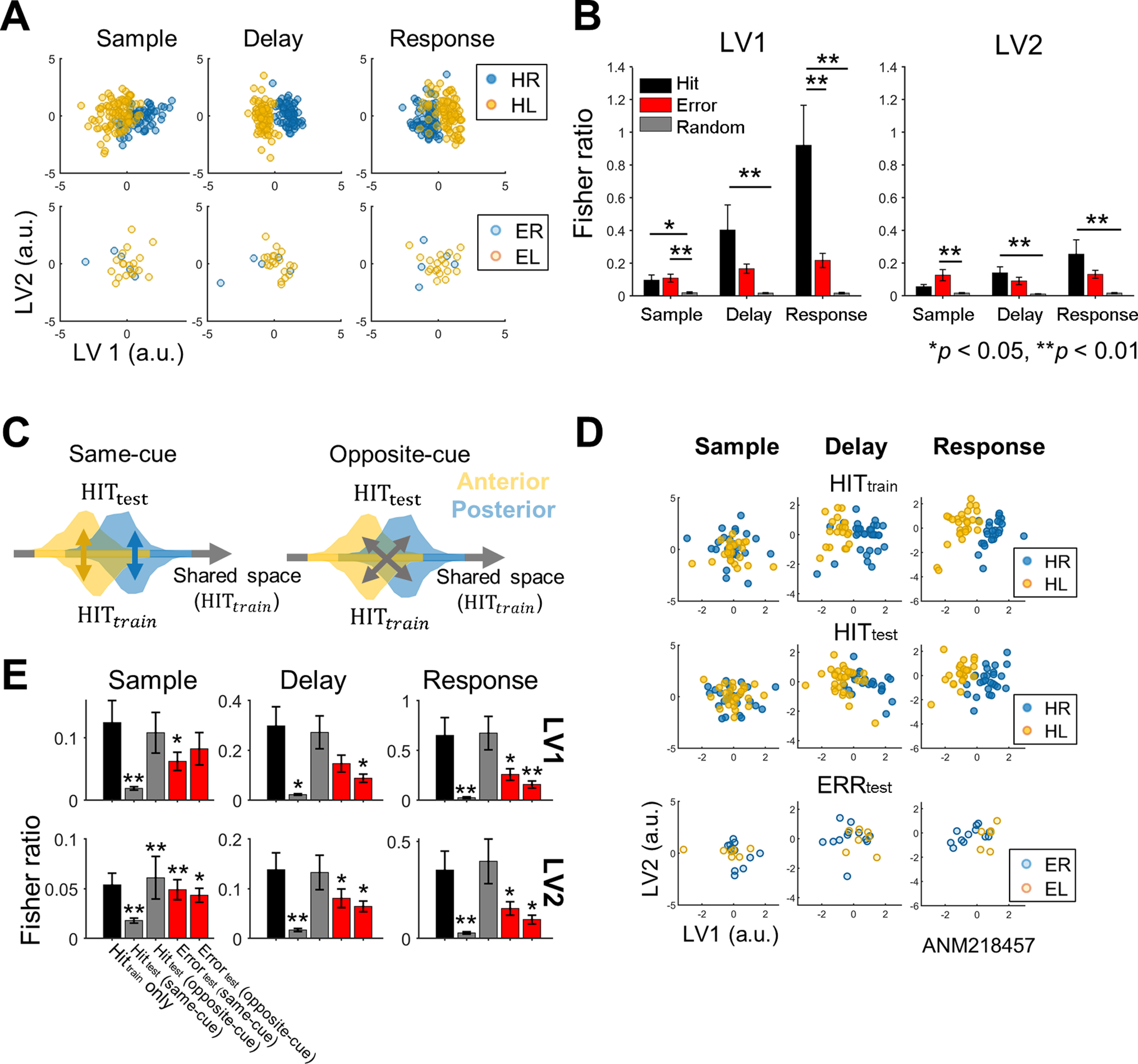
Neural representations of task-relevant information in the shared space of ALM neurons. ***A***, Examples of the task-related information representation in the shared space composed of the latent variables 1 and 2 (LV1 and LV2; top: hit trials, bottom: error trials). Each dot represents the 2D values of the latent variables resulting from the FA of the firing rates of ALM population at each trial. In the hit trials, the latent variables (especially LV1) distinctly represented the target direction information (HR or HL) in all the periods, which became less apparent in the error trials. ***B***, The FR between the two groups of the values corresponding to each target direction was calculated for each latent variable (LV1 and LV2), and compared among the hit, error and randomly shuffled trials (ANOVA, *p <* *0.05, **p <* *0.01, Bonferroni-corrected *post hoc t* test). Randomly shuffling was performed for the hit trials. Error bars, SEM across sessions. ***C***, The schematic diagram for illustrating the testing of consistent emergence of task-related information in the shared space (for detailed descriptions, see Materials and Methods). A shared space is first built using HIT*_train_* data, followed by the projection of HIT*_test_* data onto that shared space (HIT*_train_* data: ALM neurons’ firing rate data from a part of the hit trials used for training the FA model; HIT*_test_*: ALM neurons’ firing rate data from the remaining hit trials not used for training). Whether the representation of task-related information in the shared space is consistent throughout the trials is evaluated by two distances: (1) same-cue distance (left; between the same cues) and (2) opposite-cue distance (right; between the different cues). Distance is measured by the FR between the two groups of the latent variable values corresponding to the train and test data, respectively (for LV1 and LV2 each). If the shared space consistently represents the target direction information across trials, then the same-cue distance would remain small while the opposite-cue distance would remain large between HIT*_train_* and HIT*_test_*. This test is also applied between HIT*_train_* and ERR*_test_*, where ERR*_test_* indicates ALM neurons’ firing rate data from the error trials. ***D***, Examples of the task-related information represented in the shared space. As a standard, HIT*_train_* was projected onto the shared space built using the same data of HIT*_train_*(top). Each dot represents the projection outcome in each trial. HIT*_test_* and ERR*_test_* were projected onto the shared space constructed by HIT_train_, respectively (middle and bottom). ***E***, The same-cue and opposite-cue distances measured by the FR for each latent variable (LV1 and LV2) in each period. First, the opposite cue distance using HIT_train_ only was measured as the standard distance value (black). Then, the same-cue and opposite-cue distances were measured for HIT_test_ (gray) and ERR_test_ (red), respectively. Note that the opposite-cue distance using HIT_train_ only was measured by the FR of HIT_train_ on the shared space built using the same HIT_train_. Each of the same-cue and opposite-cue distances was compared with the standard distance value (one-tailed paired *t* test), *p <* *0.05, **p <* *0.01. Error bars, SEM across sessions.

To investigate whether ALM neuronal population represents task-related information together, we estimated a 2D shared space of the firing rates of ALM neuronal populations using FA. We could identify latent variables (i.e., factors) that best describe covariance matrix between population of neurons through FA. Since two principal components capture over the majority of variance (>60%) of data in every period of the hit and error trials, the number of latent variables was fixed to two. Although the shared space was estimated solely from neuronal data in an unsupervised way, we observed that task-related information (i.e., cued licking direction) was present in the shared space (Fig. 4*A*, top). The FR between the two clusters in the shared space formed based on the cue information (i.e., HR vs HL or ER vs EL) showed a significant difference between the hit, error, and shuffled trials (*p*s < 0.05 for every period and latent variable, one-way ANOVA; Fig. 4*B*). A *post hoc* analysis showed that the FR of the hit trials was greater than that of the shuffled trials in every period for both latent variables except in the sample period for the second latent variable. However, the FR of the error trials was greater than that of the shuffled trials only in the sample period (*p *<* *0.01, Bonferroni-corrected *post hoc t* test; Fig. 4*B*). Besides, it showed that the FR of the hit trials was greater than that of the error trials in the response period on the first latent variable (*p *<* *0.01). Thus, in the hit trials, the cue information was separately represented in the shared space, which became less distinguishable in the error trials.

Next, we tested the reliability of this representation of task-related information in the shared space via a train-and-test scheme (see Materials and Methods). We constructed the shared space using the first half of the hit trials. The second half of the hit trials and the error trials were projected on the built shared space and measured FR to test whether the directional information is still separated on the shared space. If emergent shared space has consistent axes across trials, then FR of test data would show FR values similar to those projected by the train data. The shared space built from the first half of the hit trials consistently maintained a discriminative spatial pattern for the second half of the hit trials projected onto that shared space (Fig. 4*D*, middle). In contrast, the projection of the error trials onto the same shared space did not show a discriminative spatial pattern clearly (Fig. 4*D*, bottom). Using the FR, we assessed the similarity of clustering patterns in the shared space across the hit and error trials (see Materials and Methods). Between the two clusters across the first and second halves of the hit trials corresponding to the same cue (Fig. 4*C*, left), the FR was significantly reduced compared with original FR of the first half of the hit trials for every period (*ps *<* *0.05, one-tailed paired *t* test), showing that the clusters assigned to the same cue remained largely unchanged across the hit trials (Fig. 4*E*). In contrast, the FR with the opposite cue (Fig. 4*C*, right) showed no difference from the reference value except for the second latent variable during the sample period, showing that distinct representations of licking directions were maintained across the hit trials. Between the hit and error trials (HIT*_train_* – ERR*_test_*), the FR was largely reduced in the error trials with both the same and the opposite cue (*p *<* *0.05, one-tailed paired *t* test; Fig. 4*E*), showing that task-related information in the error trial was not represented as clearly as in the hit trials. Moreover, the FR reduced more with the opposite cue than with the same cue in the delay and response periods of the error trials (*p *<* *0.01, one-tailed paired *t* test), which indicated that clustering patterns in the error trials appeared to be relatively closer to those in the hit trials if licking directions were switched.

### ALM neurons showed selectivity in the shared signals

**Figure 5.**
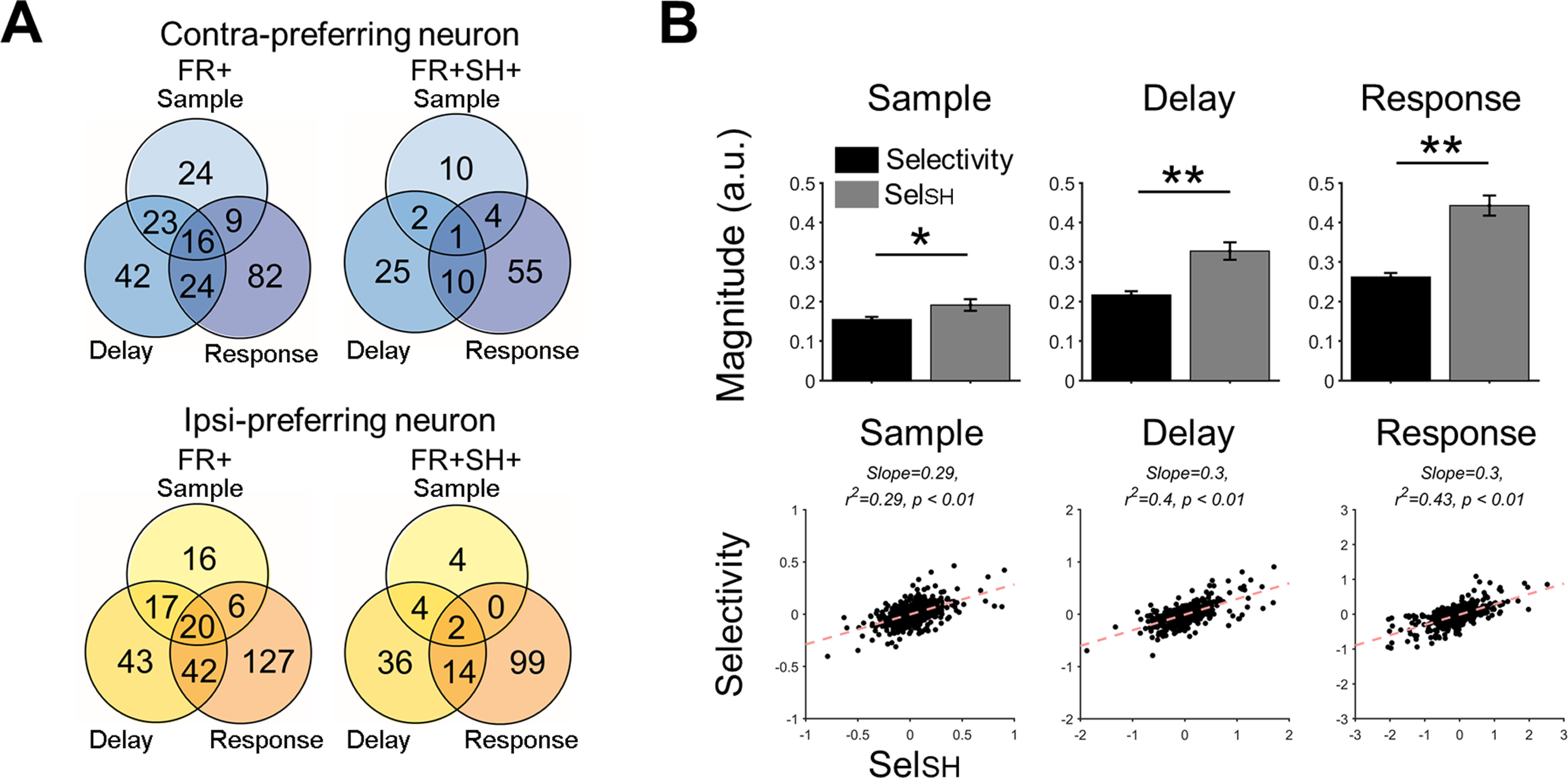
Selectivity in firing rates and shared signals of ALM neurons. ***A***, Venn diagrams of the number of neurons showing selectivity in each period. FR+ denotes the neurons that have selectivity in firing rates (top: contra-preferring neuron; bottom: ipsi-preferring neuron). FR+SH+ denotes then neurons that have selectivity in both firing rates and shared signals (for the description of the shared signal of a neuron, see Materials and Methods). ***B***, For the FR+SH+ neurons, selectivity in shared signals (Sel_SH_) is greater in magnitude than selectivity in firing rates (Selectivity) in every period (top, one-tailed paired *t* test, *p*s < 0.05 for every period). Linear regression of Selectivity against Sel_SH_ yielded significant linear fits (bottom, *p*s* *<* *0.05), with every slope <1 in each period.

In this section, we investigated how the selectivitiy of individual neurons was related to the shared space and whether such a relationship was altered for erroneous behavior. By decomposing the firing rate of a neuron into shared signals and private signals using FA (see Materials and Methods), we analyzed the shared signals that reflected how the neuronal firing rate was modulated by the shared space (see Materials and Methods). Of a total of 634 recorded ALM neurons, we observed 220 contra-preferring neurons and 271 ipsi-preferring neurons (FR+; Fig. 5*A*). Among these selective neurons, 107 contra-preferring neurons (48.6%) and 159 ipsi-preferring neurons (58.7%) also showed selectivity in their shared signals (FR+SH+; Fig. 5*A*). We focused on these FR+SH+ neurons, in which task-related information in the shared space was reflected on the firing rate. Next, we compared the magnitudes of selectivity between the firing rates and shared signals of the FR+SH+ neurons. We found that the selectivity of shared signals (Sel_SH_) was significantly greater than that of firing rates (*p*s < 0.05 for every period, one-tailed paired *t* test; Fig. 5*B*, top). A linear regression analysis with Sel_SH_ as an independent variable and that of firing rates as a dependent variable showed a significant linear relationship with slopes <1 (*p*s < 0.01; Fig. 5*B*, bottom). The result supports our assumption on a generative relation of firing rates from latent variables that the selectivity of a single neuron may be related to the shared space composed by population activity.

From the observed changes in firing rates (Fig. 2*A*,*B*) and latent variable patterns (Fig. 4*A*,*D*) across the hit and the error trials, we examined how firing modulation of selective neurons was altered during the error trials. In the perspective of a generative model (FA), if latent variables in the error trials represent licking direction contrary to the direction that they should have represented while the generative relationship described in the factor loading matrix remains unchanged, the shared signal of selective neurons that are generated from the latent variables should also exhibit selectivity in an opposite way to the hit trials.

**Figure 6.**
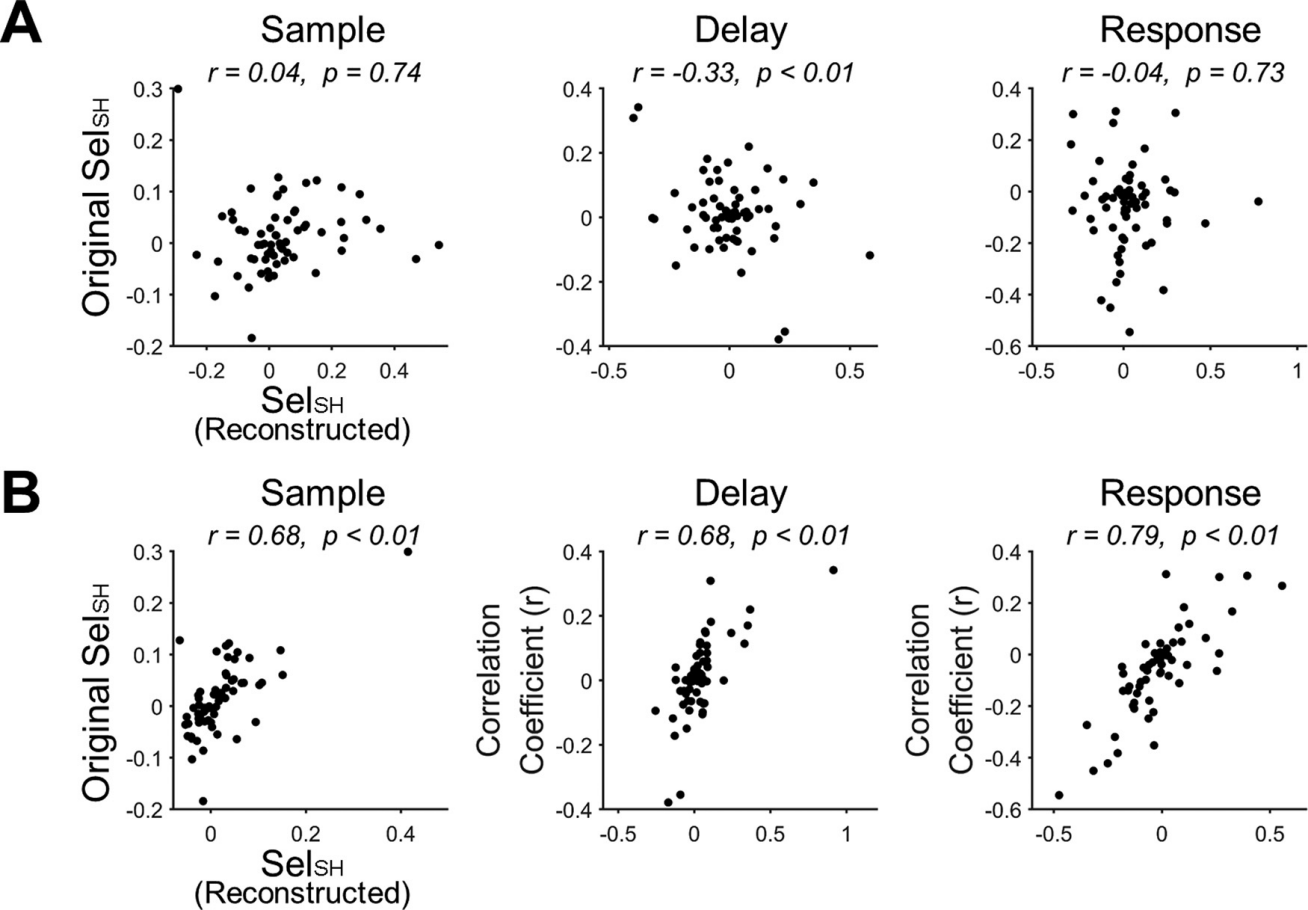
Alteration of selectivity in erroneous behavior is related to alteration of latent variables while relations between firing rates and latent variables are unchanged. ***A***, The scatter plots of reconstructed Sel_SH_ and original Sel_SH_ in the hit trials. Each dot denotes each session. Reconstructed Sel_SH_ was the selectivity of the shared signals reconstructed by the loading matrix (*U*) obtained from the hit trials and latent variables (*z*) obtained from the error trials. A significant correlation was observed between reconstructed Sel_SH_ and original Sel_SH_ only in the delay period (*p *<* *0.01), where the correlation coefficient was negative (*r* = −0.33). The negative correlation indicates that *z* in the error trials were reversely formed, thus generating altered selectivity (see the text for more details). ***B***, The scatter plots of reconstructed Sel_SH_ and original Sel_SH_ in the hit trials. Different from ***A***, the shared signals were now reconstructed using *U* obtained from the error trials and *z* from the hit trials. For every period, reconstructed Sel_SH_ and original Sel_SH_ were positively correlated (*p*s < 0.01).

Since this collectively reversed firing modulation indicated that the altered selectivity of ALM neurons in the error trials might be driven by changes in the shared space, rather than an independent change of modulation in individual neurons, we analyzed the possible changes in the generation of shared signals from latent variables during the error trials. On one hand, when we generated shared signals from latent variables using the error trials while keeping the factor loading matrix (*U*), their selectivity became uncorrelated with their original selectivity obtained from the hit trials in the sample and response periods, or even negatively correlated in delay period (*r* = −0.33; Fig. 6*A*). On the other hand, if we generated shared signals using *U* estimated from the error trials while keeping latent variables, their selectivity was positively correlated with their original selectivity in every period (*r *=* *0.68 for the sample, *r *=* *0.68 for the delay, and *r *=* *0.79 for the response period; Fig. 6*B*). Hence, we confirmed that latent variables were altered during the error trials rather than overall generative relationships from latent variables to shared signals.

### ALM neuronal selectivity is correlated with communality

**Figure 7.**
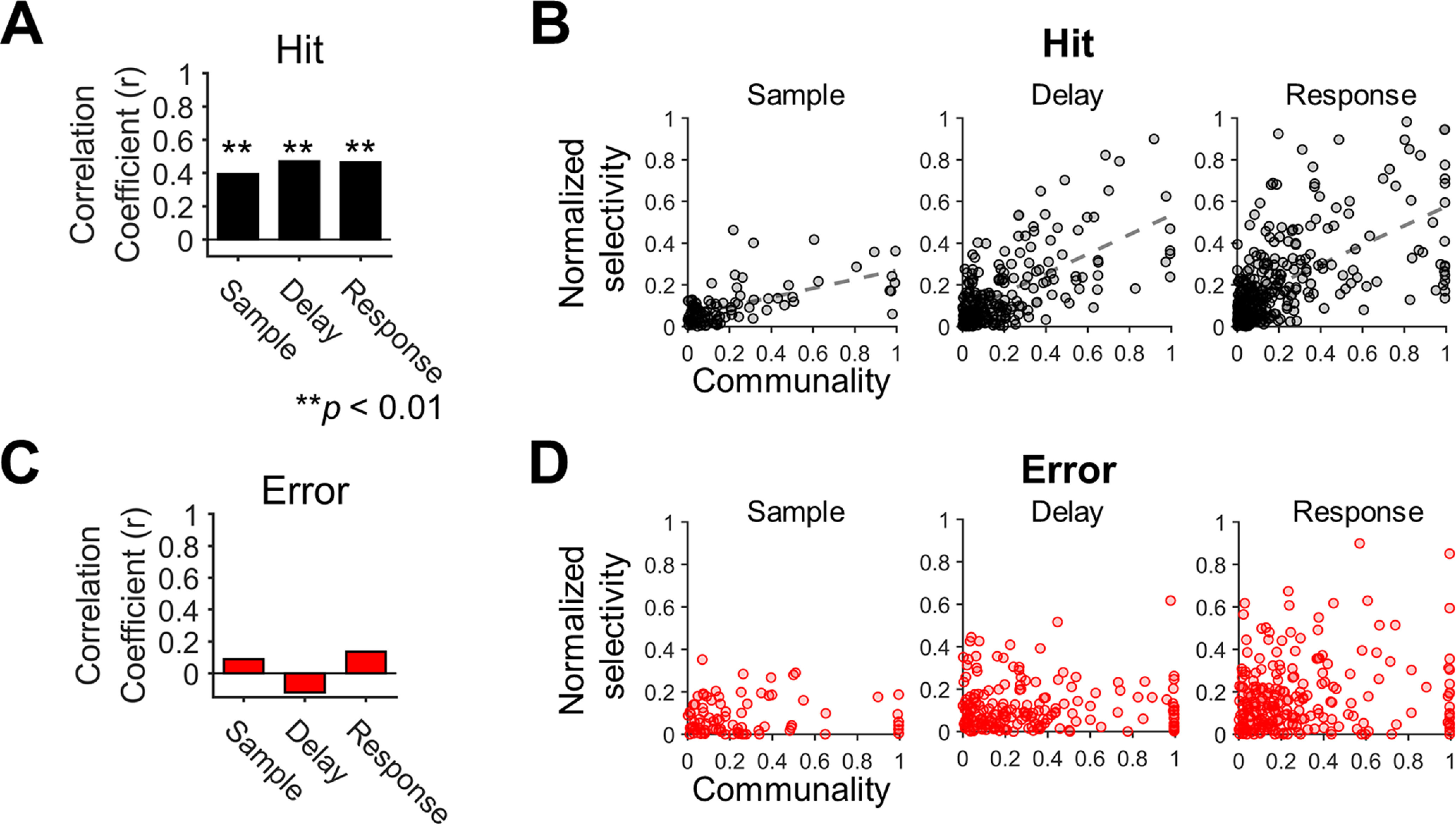
Selectivity of individual neurons is positively correlated with their communality to the shared space. ***A***, Correlations between communality and selectivity in the hit trials. The communality and selectivity across individual neurons were positively correlated in every period (*r*, Pearson’s correlation coefficient, **p <* *0.01). ***B***, The scatter plots of communality and selectivity across individual neurons in the hit trials. The dashed lines indicate significant regression lines obtained from linear regression (*p*s < 0.01). Each circle reflects a single neuron. Note that selectivity was normalized before calculating correlations to compare the differences between neurons regardless of the session. ***C***, Correlations between communality and selectivity in error trials. No significant correlation was observed in any period (*p*s > 0.1). ***D***, The scatter plots of communality and selectivity across individual neurons in the error trials. Linear regression revealed no significant linear relationships between communality and selectivity (*p*s > 0.1). Each circle reflects a single neuron.

For each neuron, we measured communality to determine how well the neuron’s firing rate was accounted for by the first two latent variables. Then, we examined a correlation between the magnitude of selectivity and that of communality of the FR+SH+ neurons. For the hit trials, we found significant positive correlations between communality and selectivity in every period (sample: *r *=* *0.39; delay: *r *=* *0.47; response: *r *=* *0.47; *p*s < 0.01 for every period; Fig. 7*A*,*B*). It revealed that the ALM neurons tended to be more selective when their firing rate modulation contributed more to the shared space. However, such linear relationships disappeared in the error trials (sample: *r *=* *0.06; delay: *r* = −0.11; response: *r *=* *0.14; *p*s > 0.05 for every period; Fig. 7*C*,*D*), implying that selective modulation of firing rates in the ALM neurons became irrelevant to their dependency on the shared space in erroneous behavior especially during movement preparation.

### Changes in selectivity between the hit and error trials were correlated with changes in communality

**Figure 8.**
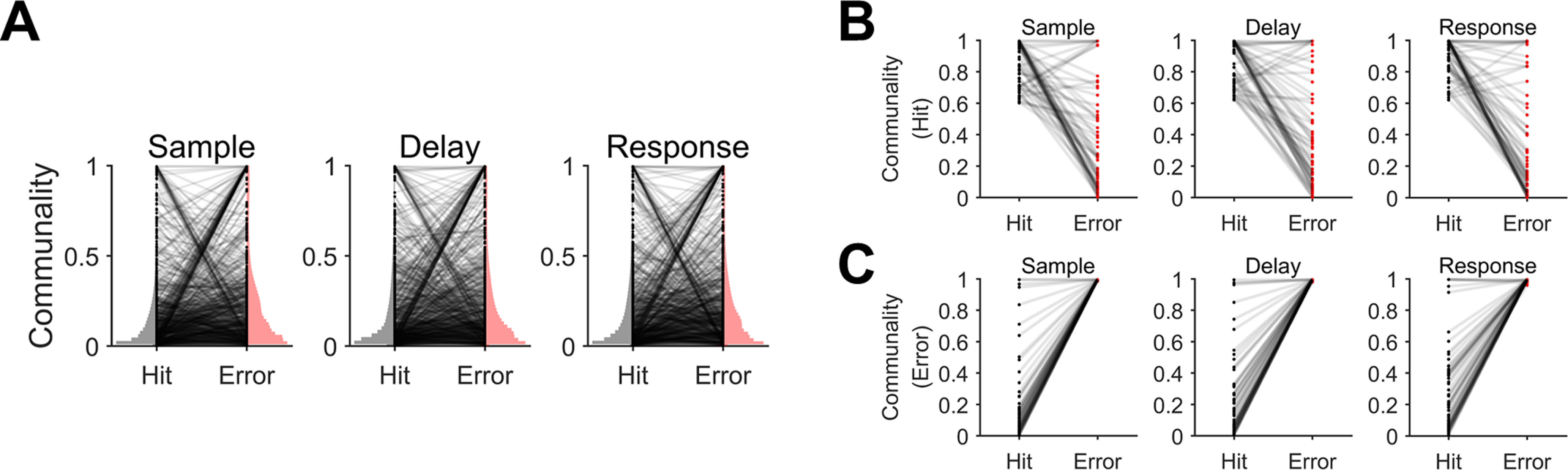
Changes in communality between correct and incorrect behavior. ***A***, Distributions of communality in the correct (hit) and erroneous (error) trials for each period (sample, delay, and response). Black dots reflect the communality of single neurons in the hit and the error trials. Gray lines connecting each pair of dots between the hit and error trial indicates communality change of the corresponding neuron between the hit and error trials. The vertically oriented shadings indicate sample distributions of selectivity for hit (gray) or error (pink) trials, respectively. The K-S test showed that the cumulative density function of communality in the hit trials was significantly smaller than that in the error trials (*p*s < 10^−4^ for every period). ***B***, Neurons with the top 10% highest communality in the hit trials significantly decreased their communality in the error trials for every period (one-tailed paired *t* test, *p *<* *0.01). Gray lines indicate communality changes from the hit to the error trials of single neurons. ***C***, Neurons with the top 10% highest communality in the error trials significantly decreased their communality in the hit trials for every period (one-tailed paired *t* test, *p *<* *0.01). Gray lines indicate communality changes from the hit to the error trials of single neurons.

To understand why correlations between selectivity and communality present in the hit trials disappeared in the error trials, we first compared overall distributions of communality between the hit and error trials. The K-S test showed that cumulative density function of communality in the hit trials was smaller than that in the error trials (*p*s < 10^−4^ for every period; Fig. 8*A*). However, we observed a similar pattern in communality changes between the hit and error trials (Fig. 7*B*) as in selectivity (Fig. 2*B*), neurons with higher communality in the hit trials tended to reduce their communality in the error trials whereas those with lower communality in the hit trials increased their communality in the error trials. To examine these observations, we examined neurons with the top 10% communality in the hit trials and found that they significantly decreased communality in the error trials and vice versa (*p*s < 0.01 for every period, one-tailed paired *t* test; Fig. 8*B*,*C*).

**Figure 9.**
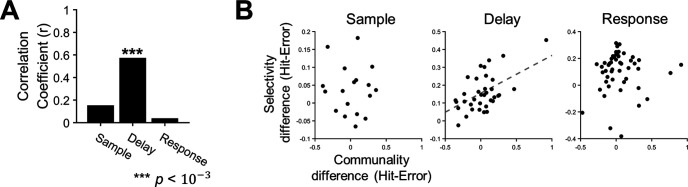
Altered selectivity of ALM neurons with motor planning error is related to altered communality. ***A***, Correlations between the mean communality change and the mean selectivity change from the hit to the error trials, where the mean was estimated over the population of neurons in each session, were calculated across sessions (*r*, Pearson’s correlation coefficient). A significant correlation was observed only in the delay period (***p < 10^–3^). ***B***, The scatter plots of the mean communality differences and the mean selectivity differences in each period. Each dot reflects each session. The dashed regression line was obtained from linear regression (*p *<* *0.01).

Upon finding this similarity between selectivity and communality, we further investigated whether neuron-level alterations in selectivity were related to those in communality. Although we did not directly estimate the shared space from the selectivity, the task-related activities would be captured in the shared space through covariance structure. Therefore, to identify a specific aspect of dependency related to behavior in the shared space, we evaluated whether each neuronal engagement on the shared space could explain the selectivity and accounted for the change in the selectivity in the error trials by changes in engagement on constructing the shared space. Specifically, we tested whether the amount of change of selectivity from the hit to error trials would be explained by that of communality. To this end, we defined a change in communality and selectivity of a neuron between the hit and error trials as Δcom = communality_Hit_ – communality_Error_ and Δsel = selectivity_Hit_ – selectivity_Error_, respectively, and performed a correlation analysis between Δsel and Δcom in each period. The result showed relatively weak but significant linear relationships between Δsel and Δcom across individual neurons (sample: *r* = 0.29, *p *<* *0.05; delay: *r *=* *0.27, *p *<* *0.01; response: *r *=* *0.33, *p *<* *10^−6^). However, when we performed the correlation analysis at the population level, where Δcom (or Δsel) was averaged over a population of ALM neurons within each session, we found a stronger correlation between Δcom and Δsel in the delay period (*r *=* *0.57, *p* <10^−3^), but not in other periods (sample: *r *=* *0.15, *p *=* *0.52; response: *r *=* *0.03, *p *=* *0.81; Fig. 9*A*,*B*). The results suggest that changes in single neurons’ selectivity underlying erroneous behavior, i.e., the decreased selectivity of originally more selective neurons and the increased selectivity of originally less selective neurons, might occur in relation to changes in those neurons’ communality, especially during a motor planning period.

## Discussion

The present study investigated neural substrates of erroneous behavior in rodents’ ALM populations during the tactile delayed-response task. Compared with correct behavior, the selectivity of individual ALM neurons was reversed. Licking direction was inadequately represented in the shared space by population, and connections of the selectivity of individual neurons to the shared space, measured by correlations between selectivity and communality, was disrupted, during erroneous behavior. Notably, average selectivity in animals changed more between correct and erroneous behavior when the corresponding average communality changed more, during the delay period. Our results suggest neural substrates of erroneous behavior in the tactile delayed-task as joint changes in the selectivity of ALM neurons at both single neuron and population levels, as well as alternation of the neuronal coupling assignment to the shared space.

One of the intriguing findings of the present study was that the single neuron-level change in selectivity between correct and erroneous behavior was highly correlated with the population-level change in communality, which was observed only in the delay period. Also, we demonstrated that highly selective neurons for correct behavior decreased their selectivity for erroneous behavior whereas less selective neurons for correct behavior became more selective for erroneous behavior. Together, significant alterations in selectivity of ALM neurons that underlie erroneous behavior were tightly linked to changes in communality during the delay period. Considering that changes of communality mean changes of the degree to which a neuron’s activity is coupled to the shared space, our results suggest that incorrect modulation of ALM neurons that are less selective during movement preparation would be engaged in causing behavioral error as supported by changes in selectivity.

Individual neuronal activities vary in part with those of other neurons, which creates “common variance” shared among a number of neurons. Existence of such shared variance among neurons enables us to find a low-dimensional space mathematically in which each dimension represents co-varying activity of a subset of neurons in the population. If the shared variance changes with the task, the task-related information would also be represented on the shared space. In this study, we confirmed that future licking direction was discriminately represented on the shared space for correct behavior but not for erroneous behavior. This implied that co-varying activity of a subset of neurons in the population was not correctly coordinated for erroneous behavior, indicating a possible error in the interaction between those neurons.

Moreover, stronger coupling of a single neuron to the shared space means that the neuron’s activity is explained more by co-varying activity of a set of neurons that share variance. It implies that a strongly coupled neuron might participate more in generating co-varying activity pattern in the shared group. As it is known that selectivity is key to movement preparation, we can assume that tight coupling of highly selective neurons sharing the same preferred direction (i.e., contra-preferring or ipsi-preferring) would be important to make correct movements. We observed that highly selective neurons showed stronger coupling to the shared space for correct behavior and that these neurons reduced their coupling as well as selectivity for erroneous behavior. Interestingly, we rather found that a different group of neurons that showed low selectivity for correct behavior became more selective for erroneous behavior along with stronger coupling. It indicates that a wrong set of neurons became more interactive during movement preparation for erroneous behavior while the originally selective neurons were not properly coordinated. Note that this wrong set of neurons partially involved selective neurons of opposite preferred direction but mostly included neurons that had been nonselective if behaved correctly. It implies that erroneous behavior might not be a consequence of wrong sampling of tactile cue, which would have increased coupling of neurons of opposite preferred direction, but rather involve more complicated processes of neuronal interactions in the ALM circuit which remains vague and needs further in-depth investigations.

Although much more work is needed to answer why changes in selectivity were correlated with changes in communality only during the delay period, we speculate possible explanations for this as follows. First, ALM neurons are involved in retaining working memory related to future licking information in the delay period. When a tactile cue is given, primary somatosensory cortex (vS1) encodes the tactile information and subsequently transfers it to ALM (Guo et al., 2014). Also, medial motor cortex (MM) is activated in the sample period, followed by the activation of ALM neurons in deep layers in the early delay period (Chen et al., 2017). Hence, ALM neurons might become more coordinated as the delay period begins, which would be likely to tighten the coupling of population activity of ALM neurons with the shared space. Second, preparatory activities of motor cortical neurons stay on the null space of movement execution to prevent muscle from evoking overt movements, thus coupling with the shared space would also be changed after go cue (Guo et al., 2014; Stavisky et al., 2017; Economo et al., 2018).

In this study, we showed that the selectivity of individual ALM neurons in mice varied with the extent to which the neurons’ firing activities were coupled to an intrinsic manifold shared by the neurons. Our finding is in line with a recent computational study, which reported that a latent state model based on recurrent neural networks could generate virtual neurons with selectivity, suggesting that the selectivity of motor cortical neurons could be the result of latent dynamics under which a population of neurons modulates their firing activities to perform a task (Michaels et al., 2016). Yet, different from in-silico studies elucidating selectivity by latent dynamics with synthetic neurons, the present study revealed that coupling to the intrinsic manifold elucidated selectivity of biological ALM neurons.

Wei and colleagues showed similar dynamical structures underlying correct and erroneous behavior at ALM population level (Wei et al., 2019). In their study, neural representations of population activity in an intrinsic manifold reached toward the opposite direction during the error trials but also hovered over intermediate areas between two possible licking directions. Consistent with these results, we found less separable representations of population activities in the error trials. However, different from this previous study’s account of licking behavior based only on neural representations of population activity in the intrinsic manifold, the present study explains behavioral outcomes produced by individual neuronal firing characteristics (selectivity) in association with latent structure (shared space).

The present study showed that the selectivity of individual ALM neurons could be partially explained by the extent to which their firing activities were coupled to the intrinsic manifold where the task-relevant information (i.e., licking direction) was manifested (Figs. 7, 9). This new account of selectivity may be applied to other similar neuronal activities found in many brain areas such as preferred directions (Georgopoulos and Ashe, 2000; Omrani et al., 2017) as well as other types of selectivity associated with various sensorimotor and cognitive tasks (Rigotti et al., 2013; Amedi et al., 2017; Banerjee and Long, 2017).

Although biological implications of the shared space in the motor cortex remain elusive, many studies have attempted to gain insights from the analysis of the shared space regarding task-relevant neuronal population dynamics. For instance, studies have shown that the alignment of an intrinsic manifold of wide-scale motor cortical neurons occurs in the course of task learning, and the latent space becomes consolidated across neurons after learning (Ganguly et al., 2011; Koralek et al., 2012, 2013; So et al., 2012; Wander et al., 2013; Clancy et al., 2014; Gulati et al., 2014). Also, a recent study suggests that anatomically separated cortical areas interact with each other through the latent spaces (Semedo et al., 2019). Multiple brain regions are reportedly involved together with ALM in the performance of the tactile delayed-response task, including vS1, MM, thalamus, and cerebellum, implying that a large-scale shared variance may emerge across multiple brain regions after learning to perform the task (Li et al., 2016; Allen et al., 2017; Chen et al., 2017; Guo et al., 2017; Gao et al., 2018).

Dynamics underlying ALM selectivity can be described by a network model such as a discrete attractor model (Inagaki et al., 2019) and possibly elucidate how erroneous behavior occurs more precisely. But it is difficult to extend the network model to incorporate all inputs to ALM. On the other hand, low-dimensional projection can effectively represent the task-relevant variance of ALM neurons driven by input signals to ALM, because the projection methods such as FA capture shared variance across neurons evoked by recurrence and input signals. For example, the trajectory on the low-dimensional space of ALM showed ramping patterns similar to those elicited by a ramping input from thalamus (Li et al., 2016; Inagaki et al., 2019). Thus, in future studies, additional investigations are required to understand what aspect of neural network dynamics is manifested the shared space. FA captures latent variables using covariance among neurons, thus prominent inputs to ALM would be reflected on the covarying activities of many ALM neurons, which is represented by latent factors. Considering that VM/VAL of thalamus drives ALM dynamics, the shared space of ALM populations inferred by FA might represent a subspace in which neuronal dynamics temporally evolve by strong thalamic inputs (Guo et al., 2017). If thalamic feedback through the thalamocortical loop falsely draws temporal growth of ALM activities to the fixed points corresponding to opposite licking direction, then neural representation in the shared space would also change accordingly. In this scheme, the selectivity of each ALM neuron would be altered depending on how much each neuron is weighted by thalamic inputs, which would be described by communality in FA.

## Synthesis

Reviewing Editor: Bong-Kiun Kaang, Seoul National University Decisions are customarily a result of the Reviewing Editor and the peer reviewers coming together and discussing their recommendations until a consensus is reached. When revisions are invited, a fact-based synthesis statement explaining their decision and outlining what is needed to prepare a revision will be listed below. The following reviewer(s) agreed to reveal their identity: Jeongjin Kim.

Reviewer 1.

This is an interesting study for understanding neural correlates for erroneous behaviors.

They sophisticatedly analyzed the firing pattern, specificity for tactile behavioral decision in an individual neuron or population neurons, and the interaction between single neuron and population neurons.

They newly found that proper association with the shared space is critical for regulating erroneous behaviors in ALM. It will broaden our mechanical insights underlying neural mechanisms of accurate planning of our actions.

It seems that the manuscript would be better if the authors share an edited version with comments as below.

1. What is the physiological meaning of ’shared space’ for erroneous behavior?

It would be nice if the authors can add the putative role of the phenomena (neurons’ coupling or decoupling to the shared space) for tactile behavioral decisions in the discussion.

2. Overall, the figure legends for each figure should be changed to describe more details for better understanding.

-In figure 2B, it would be good if you can add more details for describing the differences between the three graphs for the top and three graphs for the bottom.

-In figure 9, the description for figure B is not clear.

3. Authors use various analysis methods for getting conclusions. ex) factor analysis, defining reverse firing modulation

- The authors describe the details for the analysis method in the method section, so they briefly mention ’see method section’ in the results.

- However, it would be good to better understand if they add more explanation why they use this method and mention again the short definition of their parameters for analyzing in the result section.

4. In discussion, please check line 442 and change the first capital

Reviewer 2.

In this study, the authors attempt to study the neural substrates of erroneous behavior in the ALM region of rodents during a tactile delayed-response task. They successfully show that selectivity of many neurons is reversed during error trials, and then (in my view unsuccessfully) attempt to claim that the relationship between single-neuron selectivity and population properties, such as the relationship between selectivity and communality with the shared space, are also factors involved in determining whether a trial is correct or erroneous.

Major points:

1- Throughout the manuscript, there are comparisons between correct and error trials that do not consider the lower number of error trials as a factor to consider. This makes it hard to assess whether the differences observed in correct and error trials are the result of lower power in the error trial analyses or real differences. A solution would be to subsample correct trials to match the number of error trials and replicate the analyses where necessary.

2- Also, throughout the manuscript, the discussion of neuronal selectivity and population properties (like the shared space) are treated as independent objects of study, when in fact they are definitionally not... neuronal activities and the covariance structures of the populations they form, compose the population properties studied here. Task-related activity and selectivity are directly related to the covariance of the population across trials. So these single-cell and population-level properties are not independent and should not be studied under that assumption.

Minor points:

112: “A spike was extracted from the filtered trace by visual inspection with a spike width calculated as a tough-to-peak interval in the average spike waveform.”

Can you clarify what you mean here by “visual inspection", and how the spike width relates to this visual inspection? I doubt this was manually sorted, so some form of automatic threshold must have been used. It would be good to specify more clearly what was done (also there is a typo in 113: it’s trough)

129: I am unable to find that equation in the Inagaki 2018 paper that it supposedly came from. Could you explain in more detail the method? Why the 2x? are the max values the max during the period analyzed only? Or for the whole trial? In Inagaki’s study they use the normalized FR values to do a time-resolved analysis, by analyzing each time point independently. Did you also do that here? Or is this analysis only for whole-period analysis?

134: Normalization is used to generalize across sessions? (as mentioned in the manuscript), or to generalize across neurons, regardless of the session?

Fig 2b could be labeled for the top and bottom in the figure.

159: What criteria were used by Inagaki to show that 2 dimensions represent “well” the data? Explained X% of variance? If so, please specify this criterion at this point. Also, at which trial epoch is this true? Are 2 dimensions equally good to explain variance in all 4 periods? If not, why restrict it to 2?

301: “indicating that selectivity changes between the hit and error trials were not random but rather exhibited a pattern.” I am not sure this analysis suggests this. The results are consistent with noisy selectivity. I statistical test would be useful to assert this pattern. For instance, you could show that the cell-by-cell changes are significant (i.e. by comparing the error trial selectivity to the distribution of correct trial selectivities computed by randomly subsampling correct trials to the number of error trials). My guess is that the majority of the changes will come up as non-significant, and the trend of increasing vs decreasing will not be obvious. Another thing to consider here is the ceiling effect... if selectivity cannot be much higher due to firing rate ceilings and floors, then extreme selectivity values will become the ceiling, and the natural trend will be to decrease (if there is any change).

Fig 3b (and rest of figures): please state which period is being analyzed (pre, sample, delay, response?)

Fig 4a,b: Something doesn’t add up to me in these 2 figures. The FR is a measure of how separated are the clusters along each of the LVs, but the clusters shown in Fig4a look very overlapping along the LV2, especially in the delay and response periods, whereas along LV1 they look very separated. However, the values of FR in Fig4b do not reflect these observations. Please help clarify.

331: Perhaps because you have such a low number of values to compare?

370: How can the selectivity increase here? The measure of selectivity used here does not consider signal variance (which is reduced by the projection onto the LVs), but the mean differences should not change. Is there any intuition behind this finding that could be explained here?

385: “rather than an independent change of modulation in individual neurons,” I think there is a misunderstanding here. Independent change of the modulation of individual neurons indeed could drive a change in the shared space (which is generated by the activity of individual neurons).

403: “It revealed that the ALM neurons tended to be more selective when their firing rate modulation depended more on the shared space.” I would rather interpret this result as revealing that ALM neurons with more selectivity tend to contribute more to the shared space.

405: Is this result on LVs obtained in error trials? Or is this using the correct trial derivations? If the former is true, then this result may reflect more variance in the low-powered signal. If the latter is true, it is hard to compare, since the shared spaces change between correct and error trials, as shown in Fig6.

420: Same comment as for line 301 above.

477: “Our results suggest neural substrates of erroneous behavior in the tactile delayed-task as joint changes in the selectivity of ALM neurons at both single neuron and population levels, as well as alterations in connections between selectivity and the shared space of populations.” The former is true. But the latter I don’t think was shown (as a factor that is separate from the changes in selectivity).

459: “our results suggest that incorrect modulation of ALM neurons that should have been less activated during movement preparation would cause behavioral error.” This is true, but just showing the change in selectivity would have sufficed for this claim. The other analyses, in my view, add little to no information regarding this conclusion.

## Author Response

Reviewer 1

This is an interesting study for understanding neural correlates for erroneous behaviors.

They sophisticatedly analyzed the firing pattern, specificity for tactile behavioral decision in an individual neuron or population neurons, and the interaction between single neuron and population neurons.

They newly found that proper association with the shared space is critical for regulating erroneous behaviors in ALM. It will broaden our mechanical insights underlying neural mechanisms of accurate planning of our actions.

It seems that the manuscript would be better if the authors share an edited version with comments as below.

(Summary of Revision) We appreciate the reviewer for the overall appreciation of our study. According to the reviewer’s comments below, we have added more details and interpretations about the methods and results. Also, we have added discussion regarding the interpretation of the shared space and how neuronal coupling is changed for erroneous behavior.

1. What is the physiological meaning of ’shared space’ for erroneous behavior?

It would be nice if the authors can add the putative role of the phenomena (neurons’ coupling or decoupling to the shared space) for tactile behavioral decisions in the discussion.

Answer: We thank the reviewer for this suggestion. We have added a discussion about the putative physiological meaning of the shared space in the discussion section (line 504-532).

This paragraph is newly added:

"Individual neuronal activities vary in part with those of other neurons, which creates “common variance” shared among a number of neurons. Existence of such shared variance among neurons enables us to find a low-dimensional space mathematically in which each dimension represents co-varying activity of a subset of neurons in the population. If the shared variance changes with the task, the task-related information would also be represented on the shared space. In this study, we confirmed that future licking direction was discriminately represented on the shared space for correct behavior but not for erroneous behavior. This implied that co-varying activity of a subset of neurons in the population was not correctly coordinated for erroneous behavior, indicating a possible error in the interaction between those neurons.

Moreover, stronger coupling of a single neuron to the shared space means that the neuron’s activity is explained more by co-varying activity of a set of neurons that share variance. It implies that a strongly coupled neuron might participate more in generating co-varying activity pattern in the shared group. As it is known that selectivity is key to movement preparation, we can assume that tight coupling of highly selective neurons sharing the same preferred direction (i.e., contra-, or ipsi-preferring) would be important to make correct movements. We observed that highly selective neurons showed stronger coupling to the shared space for correct behavior and that these neurons reduced their coupling as well as selectivity for erroneous behavior. Interestingly, we rather found that a different group of neurons that showed low selectivity for correct behavior became more selective for erroneous behavior along with stronger coupling. It indicates that a wrong set of neurons became more interactive during movement preparation for erroneous behavior while the originally selective neurons were not properly coordinated. Note that this wrong set of neurons partially involved selective neurons of opposite preferred direction but mostly included neurons that had been non-selective if behaved correctly. It implies that erroneous behavior might not be a consequence of wrong sampling of tactile cue, which would have increased coupling of neurons of opposite preferred direction, but rather involve more complicated processes of neuronal interactions in the ALM circuit which remains vague and needs further in-depth investigations.”

2. Overall, the figure legends for each figure should be changed to describe more details for better understanding.

-In figure 2B, it would be good if you can add more details for describing the differences between the three graphs for the top and three graphs for the bottom.

-In figure 9, the description for figure B is not clear.

Answer: We apologize for the lack of figure descriptions. To avoid confusion, we have divided Fig. 2B into Fig. 2B-C in the revised manuscript. Also, we have added more descriptions for all figure legends, including Fig. 9B, in the revised.

3. Authors use various analysis methods for getting conclusions. ex) factor analysis, defining reverse firing modulation

- The authors describe the details for the analysis method in the method section, so they briefly mention ’see method section’ in the results.

- However, it would be good to better understand if they add more explanation why they use this method and mention again the short definition of their parameters for analyzing in the result section.

Answer: We fully agree with the reviewer’s suggestion. Accordingly, we have added explanations and motivations for the analysis methods in the result section (line 335-336, 351-355, 369-374).

4. In discussion, please check line 442 and change the first capital

Answer: We thank the reviewer for catching typo. We have fixed “licking” to “Licking” in the revised manuscript (line 484).

-

Reviewer 2

In this study, the authors attempt to study the neural substrates of erroneous behavior in the ALM region of rodents during a tactile delayed-response task. They successfully show that selectivity of many neurons is reversed during error trials, and then (in my view unsuccessfully) attempt to claim that the relationship between single-neuron selectivity and population properties, such as the relationship between selectivity and communality with the shared space, are also factors involved in determining whether a trial is correct or erroneous.

(Summary of Responses) The reviewer made specific suggestions to enhance the quality of our manuscript. We appreciate all comments and have understood the reviewer’s concerns on our manuscripts, especially issues that could arise from the interpretation of the results and the disparity in the number of trials between the hit and error trials. To strengthen the manuscript, we have conducted additional analyses and re-verified the results in the previous manuscript by subsampling as the reviewer suggested.

Major points:

1. Throughout the manuscript, there are comparisons between correct and error trials that do not consider the lower number of error trials as a factor to consider. This makes it hard to assess whether the differences observed in correct and error trials are the result of lower power in the error trial analyses or real differences. A solution would be to subsample correct trials to match the number of error trials and replicate the analyses where necessary.

Answer: We fully agree with the reviewer’s concern, which is essential to clarify differences between correct and erroneous behavior. As the reviewer suggested, we revisited our comparisons between the correct and error trials by balancing the number of trials with subsampling. We matched the trials number between the hit and error trials by randomly subsampling trials of the hit trials. This new analysis was applied to the results depicted in Fig. 2A, Fig. 6 A-B, Fig. 7, Fig. 8A and Fig. 9. However, these figures have remained unchanged after matching the number of trials because we verified that subsampling still maintained our original results.

2. Also, throughout the manuscript, the discussion of neuronal selectivity and population properties (like the shared space) are treated as independent objects of study, when in fact they are definitionally not... neuronal activities and the covariance structures of the populations they form, compose the population properties studied here. Task-related activity and selectivity are directly related to the covariance of the population across trials. So these single-cell and population-level properties are not independent and should not be studied under that assumption.

Answer: We truly appreciate this valuable comment and fully agree with the reviewer’s point that population properties (i.e., the shared space) are not independent of individual neuronal characteristics. Although we did not estimate the shared space directly from selectivity, task-relevant neuronal characteristics such as selectivity should be related to the covariance structure of a population and consequently to the shared space. Taking into account such dependence of population properties (i.e., the shared space) on individual neuronal properties, what we have aimed to find was a specific aspect of dependency related to behavior. Since selectivity is key to movement preparation, we focused on the selectivity of individual neurons and how it was related to population properties. Although it was likely that highly selective neurons would covary with other similarly selective neurons more closely and consequently contribute more to the construction of the shared space, it would not be always the case because in some cases highly selective neurons could be less correlated with other neurons. Hence, we investigated whether a neuron’s selectivity and its engagement in constructing the shared space, which we evaluated in terms of communality in this study, varied with behavior and found that selectivity and communality would be positively correlated with each other for correct behavior but such correlations disappeared for erroneous behavior (Fig. 7). No correlation between selectivity (individual neuronal properties) and communality (coupling to population properties) in the error trials supports that the dependency we investigated here was not always present and rather behavior-dependent.

We agree that the lack of explanation about our approach above was problematic in the previous manuscript, and thus we have now clarified this point in the result section as follows (line 460-466):

"Although we did not directly estimate the shared space from the selectivity, the task-related activities would be captured in the shared space through covariance structure. Therefore, to identify a specific aspect of dependency related to behavior in the shared space, we evaluated if each neuronal engagement on the shared space could explain the selectivity and accounted for the change in the selectivity in the error trials by changes in engagement on constructing the shared space.”

Minor points:

3. 112: “A spike was extracted from the filtered trace by visual inspection with a spike width calculated as a tough-to-peak interval in the average spike waveform.”

Can you clarify what you mean here by “visual inspection", and how the spike width relates to this visual inspection? I doubt this was manually sorted, so some form of automatic threshold must have been used. It would be good to specify more clearly what was done (also there is a typo in 113: it’s trough)

We apologize for the lack of descriptions in spike sorting. We have used a public dataset from Svoboda laboratory, whose spike data is sorted with manual spike sorting process as described in the study of Guo ZV et al., 2014. Briefly, they clustered spike using automated software (UltraMegaSort2000, (Fee et al., 1996; Hill et al., 2011)) and sorted manually with a MatClust due to multiple units were unresolved after using UltraMegaSort2000 (Karlsson and Frank, 2008). They saved cluster that could be separated from the background and again calculated false alarm rate (FA) by the inter-spike interval (ISI) distribution. If units too frequently fired so that violated the absolute refractory epoch, then they were excluded units (criteria: 0.5% events with ISI < 2.5 ms; a few Fast-Spiking neurons with FA>0.5%). Also, they calculated miss rate by inspecting raw voltage traces during randomly selected trials (total 12 trials). If units did not fire in so excessive misses occurred, then units were excluded. We have included the reference (Guo ZV et al., 2014) and a detailed process of spike sorting in the revised manuscript as follow (line 114∼117).

"Units with spike width < 0.35ms were defined as fast-spiking GABAergic (FS) neurons (196/2,420) and units with spike width > 0.45ms as putative pyramidal neurons (2,135/2,420). Units with intermediate values (0.35-0.45ms) were excluded from our analyses (89/2,420).”

Also, thank you for informing us the typo and we have corrected it in the revised manuscript (line 113).

4. 129: I am unable to find that equation in the Inagaki 2018 paper that it supposedly came from. Could you explain in more detail the method? Why the 2x? are the max values the max during the period analyzed only? Or for the whole trial? In Inagaki’s study they use the normalized FR values to do a time-resolved analysis, by analyzing each time point independently. Did you also do that here? Or is this analysis only for whole-period analysis?

Answer: We apologize for insufficient description. We divided the difference in firing rates between HR and HL trials by the sum of the maximum firing rates during HR and HL trials, resulting in most values in a range between -0.5∼0.5. We then multiplied the result by 2 to rescale the selectivity values between -1∼1. The maximum values in the denominator of eq. (1) in the manuscript are only for the period under consideration (sample, delay and respond period each). We used a mean firing rate in the period (averaged across time points within the period) to calculate the selectivity. We have added related information in the revised manuscript (line 139∼144).

5. 134: Normalization is used to generalize across sessions? (as mentioned in the manuscript), or to generalize across neurons, regardless of the session?

Answer: We apologize for the insufficient explanations. Normalization was used to generalize across neurons regardless of the session. We have clarified it in the revised manuscript (line 138-144).

6. Fig 2b could be labeled for the top and bottom in the figure.

Answer: We thank the reviewer for the suggestion. When we revisited Fig 2b, we noticed that the description of “top 10% selectivity” could make the reader confused with “top figure” or “bottom figure”. So, we have divided Fig. 2B into Fig. 2B and Fig. 2C for clarity in the revised manuscript. Likewise, we have divided Fig. 8B into Fig. 8B and Fig. 8C in the revised manuscript.

7. 159: What criteria were used by Inagaki to show that 2 dimensions represent “well” the data? Explained X% of variance? If so, please specify this criterion at this point. Also, at which trial epoch is this true? Are 2 dimensions equally good to explain variance in all 4 periods? If not, why restrict it to 2?

Answer: In this study, we have analyzed across-trial covariance structure to extract a low-dimensional shared space. Inagaki and colleagues found 2 dimensions capturing over 60% across-trial variance. Similarly, we found that 2 dimensions could explain over 60% across-trial variance in all the sample, delay and response periods both in the hit and error trials. Note that we did not analyze a pre-sample period. We added this explanation in the revised manuscript (line 170∼181).

8. 301: “indicating that selectivity changes between the hit and error trials were not random but rather exhibited a pattern.” I am not sure this analysis suggests this. The results are consistent with noisy selectivity. I statistical test would be useful to assert this pattern. For instance, you could show that the cell-by-cell changes are significant (i.e. by comparing the error trial selectivity to the distribution of correct trial selectivities computed by randomly subsampling correct trials to the number of error trials). My guess is that the majority of the changes will come up as non-significant, and the trend of increasing vs decreasing will not be obvious. Another thing to consider here is the ceiling effect... if selectivity cannot be much higher due to firing rate ceilings and floors, then extreme selectivity values will become the ceiling, and the natural trend will be to decrease (if there is any change).

Answer: We appreciate this critical point. The purpose of the analysis on changes in selectivity was to examine whether the selectivity of neurons, which were significantly selective in the hit trials, changed in the error trials and vice versa, rather than examining the selectivity change of every neuron. In the previous manuscript, we analyzed the top 10% selective neurons only. But in the revised analysis, we have broadened the analysis into every selective neuron. Specifically, we collected 2 sets of neurons having selectivity in either the hit or error trials. Then, for each set, we examined cell-by-cell changes in selectivity from the hit to error trials or from the error to hit trials using the one-tailed paired t-test, as suggested by the reviewer. In the first set of neurons that were selective during the hit trials, we found that the selectivity was significantly decreased from the hit to error trials (ps < 0.01 for every period). This decreased selectivity could be inferred from Fig. 1E, which shows that the firing rates of selective neurons in the hit trials became similar for preferred or non-preferred stimuli. Also, for the second set of neurons that were selective during the error trials, the selectivity was significantly decreased from the error to hit trials (ps < 0.05 for every period). Thus, we have concluded that neurons selective during the hit trials decreased their selectivity in the error trials and neurons selective during the error trials also decreased selectivity in the hit trials. We have added these additional results in the revised manuscript (line 322-325):

"We found that neurons selective during the hit trials decreased their selectivity in the error trials (ps < 0.01 for every period, one-tailed paired t-test) and neurons selective during the error trials also decreased selectivity in the hit trials (ps < 0.05 for every period, one-tailed paired t-test).”

Also, we have clarified this result in the revised manuscript by modifying the corresponding sentence in the revised manuscript (line 326-329):

the previous sentence,

"indicating that selectivity changes between the hit and error trials were not random but rather exhibited a pattern.”

now reads,

"indicating that those neurons that were significantly selective during the hit trials decreased selectivity during the error trials, and those neurons that were significantly selective during the error trials also decreased selectivity during the hit trials.”

9. Fig 3b (and rest of figures): please state which period is being analyzed (pre, sample, delay, response?)

Answer: We appreciate this comment and have specified the period being analyzed in Fig. 3B in the revised manuscript.

10. Fig 4a,b: Something doesn’t add up to me in these 2 figures. The FR is a measure of how separated are the clusters along each of the LVs, but the clusters shown in Fig4a look very overlapping along the LV2, especially in the delay and response periods, whereas along LV1 they look very separated. However, the values of FR in Fig4b do not reflect these observations. Please help clarify.

Answer: We agree that the clusters along LV2 were much more overlapped than those along LV 1, especially in the delay and response periods, although the FR of the clusters along LV2 was greater than that of the randomly generated clusters with complete overlap (Fig. 4B). A confusion between Fig. 4B and A could arise from differences in the y-axis scale between LV1 (0 ∼ 1.4) and LV2 (0 ∼ 0.5) in Fig. 4B. Thus we have equalized the y-axis scale of Fig. 4B so as to reflect more clearly the difference in clustering between LV1 and LV2 illustrated in Fig. 4A.

11. 331: Perhaps because you have such a low number of values to compare?

Answer: We appreciate this important question. We have subsampled the hit trials and tested if the FR of the hit trials and the FR of the randomly shuffled data were significant different. The result revealed that the significant difference still held, showing that the smaller number of values in the error trials was not a primary factor for comparison results (Bonferroni-corrected post-hoc t-test). We have found that the FR of the hit trials and the FR of randomly shuffled data were significant different in the hit trials even after matching the hit trials number to the error trials (ps < 0.05 for every period and LVs).

12. 370: How can the selectivity increase here? The measure of selectivity used here does not consider signal variance (which is reduced by the projection onto the LVs), but the mean differences should not change. Is there any intuition behind this finding that could be explained here?

Answer: The z-scored firing rate was composed of the shared signal and the private signal. Thus, the selectivity in the firing rate can be simplified as Selectivity= 〖fr〗_HR- 〖fr〗_Hl=〖SH〗_HR+〖PR〗_HR-〖SH〗_HL-〖PR〗_HL=Sel_SH+Sel_PR, where fr, SH and PR denote firing rates, shared signals and private signals, respectively. Taking an absolute value of both sides, |Selectivity|=|Sel_SH+Sel_PR |, and if the sign of Sel_PR was opposite to that of Sel_SH, then Selectivity could be smaller than Sel_SH.

13. 385: “rather than an independent change of modulation in individual neurons,” I think there is a misunderstanding here. Independent change of the modulation of individual neurons indeed could drive a change in the shared space (which is generated by the activity of individual neurons).

Answer: We appreciate this critical point. However, the shared space is estimated through neuronal covariance matrix, and independent change of modulation in individual neurons with no interactions with other neurons will be captured as an independent component, ψ. As ψ does not affect the estimation of the shared space, what the shared space in the error trials represents is still a coherent structure in the covariance matrix, rather than an independent change of the modulation of individual neurons.

14. 403: “It revealed that the ALM neurons tended to be more selective when their firing rate modulation depended more on the shared space.” I would rather interpret this result as revealing that ALM neurons with more selectivity tend to contribute more to the shared space.

Answer: We fully agree with the reviewer’s interpretation. As such, we have changed the corresponding sentence in the revised manuscript (line 439-440):

"It revealed that the ALM neurons tended to be more selective when their firing rate modulation depended more on the shared space.”

now reads,

"It revealed that the ALM neurons tended to be more selective when their firing rate modulation contributed more to the shared space.”

15. 405: Is this result on LVs obtained in error trials? Or is this using the correct trial derivations? If the former is true, then this result may reflect more variance in the low-powered signal. If the latter is true, it is hard to compare, since the shared spaces change between correct and error trials, as shown in Fig6.

Answer: This result was about LVs obtained in the error trials. We have added an explanation to clarify it in legend of Fig. 7C.

16. 420: Same comment as for line 301 above.

Answer: As our response to the comment 8 above, we have conducted a similar statistical test to see if high values of communality in the hit trials (in the error trials) were decreased in the error trials (in the hit trials). After calculating communality after subsampling the hit trials number to the error trials number, we extracted top 10% of communality. Unlike the statistical test for neurons that were significantly selective in response to the review 8, communality could not be tested for significance, so neurons with the top 10% communality were selected after randomly subsampling the hit trials. We have conducted paired t-test to compare communality in the hit and error trials. We have found that high values of communality in the hit trials were not persisted in the error trials, but significantly decreased (one-tailed paired t-test, ps < 0.01 for every period). This was also observed when we compared the values of communality in the error trials - high values of communality in the error trials were significantly decreased in the hit trials (one-tailed paired t-test, ps < 0.01 for every period).

17. 477: “Our results suggest neural substrates of erroneous behavior in the tactile delayed-task as joint changes in the selectivity of ALM neurons at both single neuron and population levels, as well as alterations in connections between selectivity and the shared space of populations.” The former is true. But the latter I don’t think was shown (as a factor that is separate from the changes in selectivity).

Answer: The reviewer raised an important issue. We agree that the direct substrate of erroneous behavior is changed selectivity, and the shared space is dependent of the selectivity. Although we did not separate the selectivity change from the factor, we indirectly ruled out possible compound effects of selectivity on the shared space by revealing distinct relationship between coupling strength and selectivity depending on the period and behavioral outcomes. First, coupling strength with the shared space could explain the selectivity only in the hit trials even after subsampling to match the trial number to that of the error trials. Second, even the selectivity during the response period is higher than the selectivity in the delay period, correlation between ∆sel and ∆com does not appear in the response period. Therefore, although we did not directly exclude the effect of selectivity on the shared space, Fig. 7 and 9 can support the connection shift between selectivity and the shared space occurring in the error trials. However, we have toned down the sentence as follows (line 489-492).

"Our results suggest neural substrates of erroneous behavior in the tactile delayed-task as joint changes in the selectivity of ALM neurons at both single neuron and population levels, as well as alternation of the neuronal coupling assignment to the shared space.”

18. 459: “our results suggest that incorrect modulation of ALM neurons that should have been less activated during movement preparation would cause behavioral error.” This is true, but just showing the change in selectivity would have sufficed for this claim. The other analyses, in my view, add little to no information regarding this conclusion.

Answer: We appreciate this point. We have revised the statement in the revised manuscript as follows (line 501-503).

"our results suggest that incorrect modulation of ALM neurons that are less selective during movement preparation would be engaged in causing behavioral error as supported by changes in selectivity.”
